# Phytochemical profiling of soybean genotypes using GC-MS and UHPLC-DAD/MS

**DOI:** 10.1371/journal.pone.0308489

**Published:** 2024-08-15

**Authors:** Shuxian Li, Mei Wang, Joseph Lee

**Affiliations:** 1 United States Department of Agriculture, Agricultural Research Service (USDA, ARS), Crop Genetics Research Unit, Stoneville, Mississippi, United States of America; 2 USDA, ARS, Natural Products Utilization Research Unit, University of Mississippi, University, Mississippi, United States of America; 3 National Center for Natural Products Research, School of Pharmacy, University of Mississippi, University, Mississippi, United States of America; National Institute of Technology Rourkela, INDIA

## Abstract

Soybean is one of the most economically important crops worldwide. However, soybean yield can be substantially decreased by many diseases. Soybean genotypes could have different reactions to pathogen infection. As a first step toward investigating the biochemical basis of soybean resistance and susceptibility to disease, phytochemicals in the seeds of 52 soybean genotypes previously reported to have different reactions to diseases of soybean rust (SBR), Phomopsis seed decay (PSD), and purple seed stain (PSS) were analyzed. Using GC-MS, a total of 46 compounds were tentatively identified which included 11 chemical groups. Among those, the major group was esters, followed by carboxylic acid, ketone, and sugar moieties. Compounds having reported antioxidant, anti-microbial, and anti-inflammatory activities were also identified. UHPLC-DAD/MS analysis indicated that there were five major isoflavone components presented in the samples, including daidzin, glycitin, genistin, malonyldaidzin, and malonylglycitin. Isoflavones have been reported to play an important role in defense from plant pathogens. Although there was variance in the isoflavone content among soybean genotypes, those with the SBR resistance *Rpp6* gene (PI 567102B, PI 567104B, PI 567129) consistently exhibited the highest concentrations of daidzin, glycitin, genistin, and malonyldaidzin. The SBR resistant genotype, PI 230970 (*Rpp2*) had the greatest amount of genistin. The SBR resistant genotype, PI 200456 (*Rpp5*) resistant genotype uniquely contained glycitein, a compound that was absent in the other 51 genotypes examined. A PSD-resistant genotype PI 424324B had nearly four times the amount of stigmasterol as PI 556625, which was susceptible to SBR, PSD, and PSS in our previous tests. Results of this study provide useful information for further investigation of the biochemical basis of soybean resistance to diseases. The results may also aid in selection of soybean lines for breeding for resistance to soybean rust and other diseases.

## Introduction

Soybean (*Glycine max* (L.) Merr.) is one of the most economically important crops in the world. Although soybean is native to Asia, it was introduced into North America, Europe, and later into South and Central America, and eventually to other parts of the world [1]. World soybean production was 170 million metric tons (MMT) in 1960 and increased to 398.3 MMT in 2023 (https://ipad.fas.usda.gov/cropexplorer/cropview/commodityView.aspx?cropid=2222000). Soybean produced 70.86% of the global supply of plant-based protein meal and 28.88% of the plant-based oil in the 2020/2021 market year based on the Market View Data Base of the United Soybean Board [2]. Soybean is considered essential for global food security [3]. The seeds of soybean have 8.3 to 27.9% oil content and 34.1 to 56.8% protein content depending on the soybean varieties and cultivation conditions [4]. Studies indicate that soybeans and soy-based foods offer a plethora of health advantages, in addition to being among the most abundant and cost-effective protein sources available [5]. Soybean is ideal for human and animal nutrition [6], as well as for biodiesel production [7].

Global demand for soybean production has been significantly increasing. However, soybean production can be severely impacted by many diseases. Of more than 200 reported soybean pathogens, about 35 cause major economic impacts [8]. Soybean genotypes can have different reactions to pathogen infection [9, 10]. It has been reported that soybean contains numerous bioactive phytochemicals, which include, but are not limited to, phenolic acids, flavonoids, isoflavones, saponins, phytosterols, and sphingolipids [11–13]. Although soybean phytochemicals have a positive effect on the human immune system [14], their role in the soybean genotype’s response to pathogens has not been well-studied.

As a first step toward investigating the biochemical basis of soybean resistance and susceptibility to disease, phytochemicals in the seeds of 52 soybean genotypes previously reported to have different reactions to diseases of soybean rust (SBR) caused by *Phakopsora pachyrhizi*, Phomopsis seed decay (PSD) caused by *Diaporthe longicolla*, and purple seed stain caused (PSS) by *Cercospora* spp. were analyzed and their potential value against their causal pathogens were explored. SBR is one of the most devasting foliar diseases causing yield loss of up to 90% [8]. Both PSD and PSS are seed diseases reducing seed quality and seed lot grade for marketing purposes [8].

The specific objectives of this study were (i) to identify and classify/categorize compounds in soybean seeds using gas chromatography/mass spectrometry (GC-MS) analysis; (ii) to analyze isoflavones in soybean seeds using ultra-high performance liquid chromatography/diode-array detector-mass spectrometry (UHPLC/DAD-MS); and (iii) to profile phytochemicals among soybean genotypes. Overall, the results of this study provide useful information for further investigation of the biochemical basis of soybean resistance and susceptibility to disease. The results may also aid in selection of soybean genotypes for breeding for resistance to soybean diseases.

## Material and methods

### Plant materials

Seeds of fifty-two soybean genotypes originating from eight countries (Brazil, China, India, Indonesia, South Korea, Japan, USA, and Vietnam) were used in this study. The genotype’s name, geographical origin, and references related to the genotype’s reactions to three soybean pathogens (*Phakopsora pachyrhizi*, *Diaporthe longicolla*, *Cercospora* spp.) that cause soybean rust (SBR), Phomopsis seed decay (PSD), and purple seed stain (PSS), respectively are shown in Table 1. Seeds of these genotypes were obtained from the curators of the USDA-ARS GRIN (ars-grin.gov).

**Table 1:** List of soybean genotypes, their maturity group, geographical origin, and references related to the genotype reactions to soybean pathogens used in this study.

Sample Code	Accession (PI)	Cultivar Name	Maturity Group^c^	Genotype	Origin	Reference
SBR1	587880A	Huang dou	VI	*Rpp1c*	Zhejiang, China	[30]
SBR2	200492	Komata	VII	*Rpp1a*	Shikoku, (southern) Japan	[31]
SBR3	594538A	Min hou bai sha wan dou	IX	*Rpp1b*	Fujian, (southeastern) China	[32]
SBR4	594538B	Min hou bai sha wan dou	IX	*Rpp1b*	Fujian, (southeastern) China	[33]
SBR5	230970		VII	*Rpp2*	(Unknown), Japan	[34]
SBR6	567025A	MARIF 2592	VIII	*Rpp3*	(Unknown), Indonesia	[35]
SBR7	567046A	MARIF 2627	VIII	*Rpp3*	(Unknown), Indonesia	[35]
SBR8	567039	MARIF 2618	IX	*Rpp3*	(Unknown), Indonesia	[36]
SBR9	605854B	Sample 124	V	*Rpp3*	Tuyên Quang, Vietnam	[37]
SBR10	605891A	Sample 167	V	*Rpp3*	Sơn La, Vietnam	[37]
SBR11	605865B	Sample 136	V	*Rpp3*	Lào Cai, Vietnam	[37]
SBR12	417503	Pioneira	VI	*Rpp3*	(Unknown), Brazil	[37]
SBR13	416826A	Cha sengoku 81	VIII	*Rpp3*	(Unknown), Japan	[33]
SBR14	417132	Kyushu 56	VII	*Rpp3*	(Unknown), Japan	[37]
SBR15	605885B	Sample 160	V	*Rpp3*	Lào Cai, Vietnam	[37]
SBR16	606405	Madrak	IV	*Rpp3*	(Unknown), Vietnam	[37]
SBR17	605838	Xanh si man	V	*Rpp3*	Hà Giang, Vietnam	[37]
SBR18	615437	A 9	VI	*Rpp3*	(Unknown), Vietnam	[37]
SBR19	417089A	Kuro daizu	IX	*Rpp3*	(Unknown), Japan	[33]
SBR20	567056B		VIII	*Rpp3*	(Unknown), Indonesia	[33]
SBR21	462312	Ankur	VIII	*Rpp3*	Uttar Pradesh, India/Florida, USA	[38]
SBR22	567099A	MARIF 2740	IX	*Rpp3*	East Java, (central) Indonesia	[18, 35]
SBR23	635999	DT2000	VI	*Rpp3+Rpp4*	Taiwan, China	[39]
SBR24	506764	Hyuuga	VII	*Rpp3+Rpp5*	(Unknown), Japan	[40]
SBR26	417089B	Kuro daizu	IX	*Rpp3*?	(Unknown), Japan	[33]
SBR27	605791A	Sample 55	VI	*Rpp4*	Cao Bằng, Vietnam	[37]
SBR28	459025B	Bing nan	IX	*Rpp4*	Fujian, (southeastern) China	[41]
SBR29	200456	Awashima Zairai	VIII	*Rpp5*	Shikoku, (southern) Japan	[42]
SBR30	200487	Kinoshita	VIII	*Rpp5*	Shikoku, (southern) Japan	[42]
SBR31	471904	Orba	IX	*Rpp5*	(Unknown), Indonesia	[42]
SBR32	200526	Shira Nuhi	VIII	*Rpp5*	(Unknown), Japan	[42]
SBR33	567129	MARIF 2796	IX	*Rpp6*	(Unknown), Indonesia	[37]
SBR34	567104B	MARIF 2769	IX	*Rpp6*	East Java, (central) Indonesia	[43]
SBR35	567102B	MARIF 2767	IX	*Rpp6*	East Java, (central) Indonesia	[18]
SBR36	605823		IX	*Rpp7*	Ha giang, (northern) Vietnam	[44]
SBR37	605773	Sample 36	V	Unknown	Cao Bằng, Vietnam	[37]
SBR38	567189A	Ekhabac	IV	Unknown	(Unknown), Vietnam	[33]
SBR39	224268	Asomasari	VIII	Unknown	Hyôgo, Japan	[33]
SBR40	203398	Abura	VIII	Unknown	(Unknown), Brazil	[37]
SBR41	471208	Oka Kaizu	VIII	Unknown	(Unknown), India	[37]
SBR42	606440A	VX 92	IV	Unknown	(Unknown), Vietnam	[33]
SBR43	567351B	Tu huang dou	III	Unknown	Gansu, China	[33]
SBR44	470227B		III	Unknown	Liaoning, China	[33]
SBR45	417125	Kyushu 31	VIII	*Rpp2*	(Unknown), Japan	[45]
SBR46	518671	Williams 82	III	S to SBR, PSD, PSS	Illinois, United States	[9, 18, 38]
SBR47	556625	AP 350	IV	Check^a^, S to SBR, PSD, PSS	United States	[19, 21, 46]
SBR48	587886	Bai dou	VI	S to SBR	Zhejiang, China	[30]
SBR49	605833	Sample 102	IX	S to SBR	Hà Giang, Vietnam	[38]
SBR50	424324B	KAS 333–10	V	R^b^ to PSD	Chungcheongbuk-do, Korea, South	[47]
SBR51	458130	KAS 354–8	V	R to PSD	Chungcheongnam-do, Korea, South	[47]
SBR52	399045	KLS 906	V	S to PSD	Jeju-teukbyeoljachido, Korea, South	[47]
SBR53	549020	Lu cha dou	V	R to PSD	Liaoning, China	[47]

^a^ S = susceptible; SBR = soybean rust; PSD = Phomopsis seed decay; PSS = Purple seed stain.

^b^ R = resistant;

^c^ a group of soybean genotypes defined by adaptation within certain latitudes [8].

### Chemicals and standards

**Fig 1:**
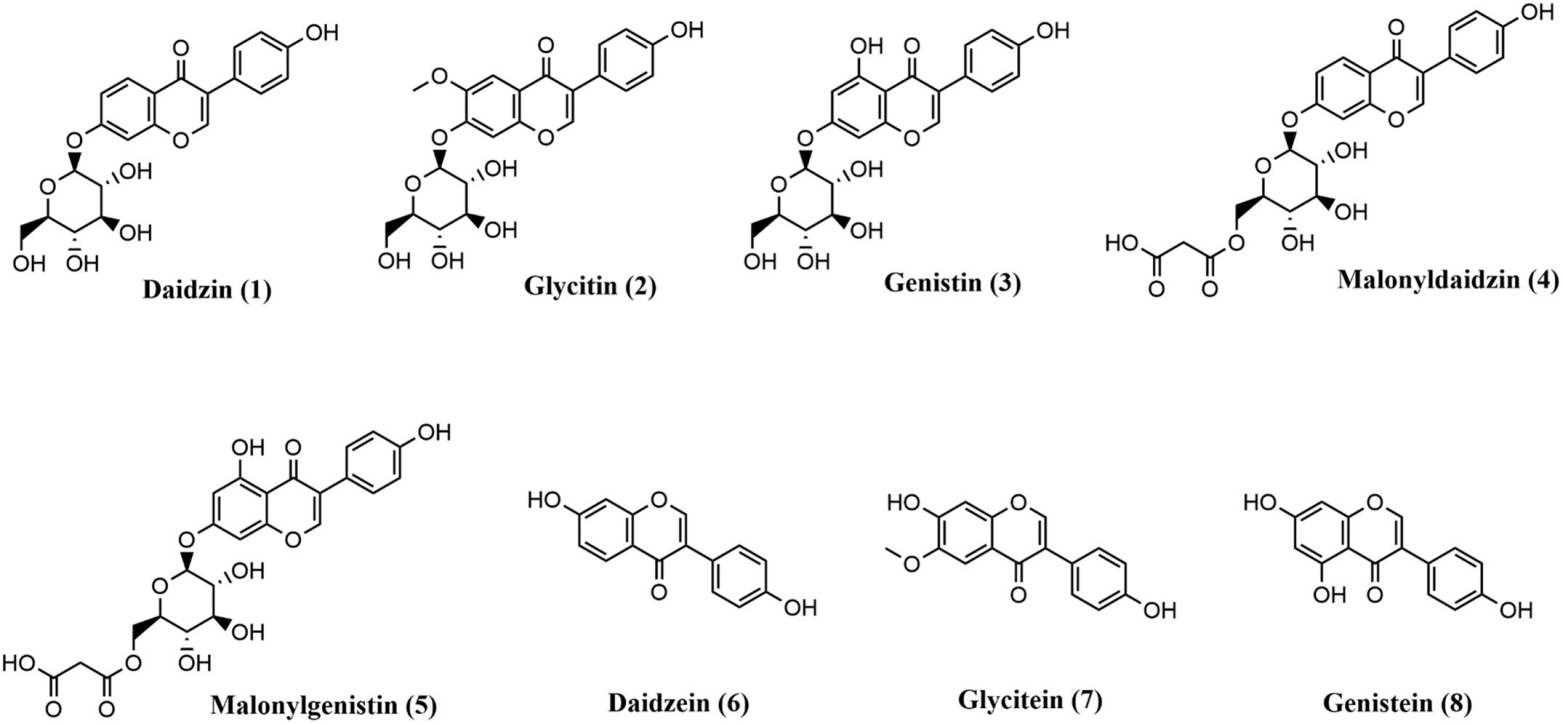
Chemical structures of the major isoflavone components in soybean seeds analyzed by LC/DAD-MS.

Chemical structures of eight primary isoflavone compounds in soybeans are shown in Fig 1. Daidzin (> 96%), daidzein (> 98%), genistin (>99%), genistein (> 99%), glycitin (>99%), and glycitein > 99%) were purchased from Indofine (Township, NJ, USA). Malonyldaidzin (> 95%) was obtained from BOC Sciences (Shirley, NY, USA). Malonylgenistin (> 98%) was obtained from AvaChem Scientific (San Antonio, TX, USA). LC-MS grade methanol, acetonitrile, and formic acid were purchased from Sigma-Aldrich (St. Louis, MO, USA). Water was purified using a Milli-Q system (Millipore, Bedford, MA, USA).

### Sample extraction method

Eight isoflavones were quantified from a total of 52 soybean seed samples. Solid soybean seeds were ground and homogenized to obtain a uniform matrix. For GC-MS analysis, 5 mL methanol was added to 100 mg of soybean seed powder and sonicated at room temperature for 60 min. For LC/DAD-MS analysis, 1.5 mL of 70% ethanol (EtOH, v/v) aqueous containing 0.1% formic acid was added to 100 mg of soybean seed powder, sonicated at room temperature for 30 min, and then centrifuged at 14,000 r/min for 10 min. The supernatant was filtered. The whole process was repeated two additional times, and then the volume was fixed to 5 mL in a volumetric flask. Both of the extraction solutions for GC-MS and LC-DAD/MS analyses were stored at 4ºC.

### Gas Chromatography-Mass Spectrometry (GC-MS) analysis

The methanolic extracts of 52 soybean seed genotypes were analyzed using an Agilent 7890 GC (Agilent Technologies, Santa Clara, CA, USA) equipped with a 7693 autosampler. Separation was achieved on an Agilent DB-5MS ultra inert column (60 m x 0.25 mm x 0.25 μm). The helium carrier gas was set to constant flow mode at 1 mL/min. The inlet was held at 260ºC and was operated in split mode with a split ratio of 50:1. The GC oven temperature was ramped from 80ºC at 3ºC/min to 125ºC, programmed at 1ºC/min to 140ºC, held for 10 min at 140ºC, then ramped at 3ºC/min to 170ºC, and finally ramped at 8ºC/min to 280ºC, where it was held for 10 min. Triplicate injections of each sample were made with a volume of 1 μL. The peak area percent was calculated as an average of the three injections. The presence/absence of each compound was defined based on the detection of the compound in the triplicate injections with a threshold of S/N > 3:1.

The mass spectral detector was an Agilent 5977A quadrupole mass spectrometer operated in the full spectral acquisition mode. The mass spectrometer was equipped with an electron ionization source, which was operated with an electron voltage of 70 eV. The ion source, quadrupole, and transfer line temperatures were set to 230, 150, and 280ºC, respectively. Data were acquired using MassHunter Acquisition software (B.07006.2704).

Data analysis was performed using MassHunter Qualitative analysis software (B.07.00).

Compound identification involved a comparison of the spectra with the NIST database (Version 2.2) using a probability-based matching algorithm.

### Ultra-high performance liquid-diode array detector/mass spectrometry (UHPLC-DAD/MS) analysis

Analysis of the isoflavone components in soybean seeds was performed on a 1290 Infinity series UHPLC system equipped with a diode array detector, binary pump, autosampler, and thermostatted column compartment. Separation was achieved using an Agilent ZORBAX Eclipse Plus C_18_ column (2.1 x 100 mm, 1.8 micron) maintained at 30ºC throughout the analysis. The mobile phase consisted of water (A) and acetonitrile (B) both containing 0.1% formic acid. The gradient elution was as follows: 0 min 10% B, 0–20 min 40% B, 20–25 min 100% B. A 5 min wash of 100% B followed each run, after which an equilibration period of 6 min with 10% B was completed. The eluent was pumped at a flow rate of 0.25 mL/min with the injection volume set at 2 μL. The DAD wavelength was set at 250 and 260 nm. The optimized wavelength for quantification of each compound was also evaluated [15]

The mass spectrometric analysis used for compound identification and confirmation was performed with an Agilent 6120 quadrupole mass spectrometry equipped with an ESI source using the following parameters: drying gas (N_2_) flow rate of 10 L/min and temperature 300ºC, nebulizer pressure 30 psi, sheath gas temperature 325ºC with a flow rate of 10 L/min, capillary voltage 3000 V, and fragmentor voltage 120 V. The data acquisition was controlled by Agilent MassHunter Acquisition Software (Ver. A.05.01) and data analysis was processed with MassHunter Qualitative Analysis and MassHunter Quantitative Analysis Software (Ver. B.10.0).

Eight standards of isoflavone components, including daidzin (1), glycitin (2), genistin (3), malonyldaidzin (4), malonylglycitin (5), daidzein (6), glycitein (7), and genistein (8) were used in the analysis. The individual stock solutions of the eight reference standards were prepared in 70% ethanol at 5 mg/mL (for those standards which have solubility issues, 70% ethanol with 10 drops of DMSO was used). These stock solutions were diluted to obtain the concentrations required for preparation of a standard working solution ranging from 0.5 to 625 μg/mL (625, 500, 400, 312, 200, 100, 75, 50, 25, 10, 5, and 0.5 μg/mL). Based on the calibration curve, the slope, intercept, and *R*^*2*^ values were calculated.

### GC-MS data analysis

The percent peak area data obtained from GC-MS analysis was combined into different groups of compounds and then exported to SIMCA-P + 13.0 software (Umetrics AB, Umeå, Sweden). With the variables in the dataset being Pareto scaled, principal component analysis (PCA) was performed. The identification with the highest probability score was taken for each compound.

## Results and discussion

### GC-MS analysis

**Fig 2:**
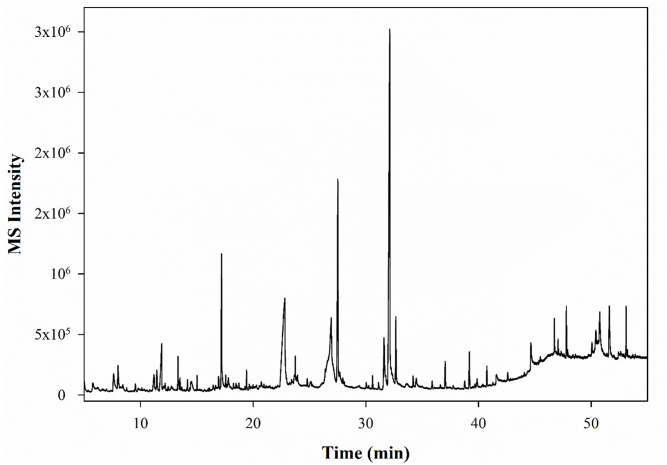
A representative total ion chromatogram of soybean seed genotype (PI230970, SBR5).

Using GC-MS analysis, a total of 46 compounds were identified based on NIST database search, retention time, and retention indices compared with literature data. A typical total ion chromatogram of one soybean genotype is shown in Fig 2. The tentative compound identification and classification results given in Table 2 and S1 Table indicated that the methanolic extract was mainly comprised of 11 chemical groups, including esters, carboxylic acids, ketones, sugar moieties, heterocyclic compounds, and phenolic compounds. Compounds having reported antioxidant, anti-microbial, and anti-inflammatory activities were identified in Table 2.

**Table 2:** Phytocompounds identified in the methanolic seed extract of 52 soybean genotypes by GC-MS.

*Rt* (min)	Compound	Chemical Group	Reported Activity	Source
5.044	Methyl pyruvate	Ester	Insulinotropic	[48]
7.763	Dihydroxyacetone	Ketone	Skin tanning	[49]
8.068	2,4-Dihydroxy-2,5-dimethyl-3(2H)-furan-3-one	Ketone	Anti-helmetic	[50]
8.254	Pyranone	Heterocyclic compound	N/A	
11.530	2-Hydroxy-γ-butyrolactone	Ketone	Insecticide	[50]
11.887	Maltol	Ketone	Flavor enhancer	[51]
12.025	l-Alanine, n-propargyloxycarbonyl-, hexyl ester	Ester	N/A	
12.168	4,5-Diamino-2-hydroxypyrimidine	Heterocyclic compound	N/A	
15.101	Serine acetate	Ester	N/A	
15.130	l-Alanine, N-methoxycarbonyl-, heptyl ester	Ester	N/A	
15.144	Butanoic acid, 2-methyl-3-oxo-, ethyl ester	Ester	Algaecide	[52]
15.316	Propanoic acid, 3-(acetylthio)-2-methyl-	Carboxylic acid	N/A	
17.211	5-Hydroxymethylfurfural	Aldehyde	Antioxidant	[53]
17.625	4-Vinylguaiacol	Phenolic compound	Antioxidant, anti-microbial, anti-inflammatory	[54]
19.440	2,3-Dimethoxyphenol	Phenolic compound	Antioxidant, anti-microbial, anti-inflammatory	[54]
22.916	Sucrose	Sugar moiety	N/A	
23.759	3,5-Dimethoxyacetophenone	Ketone	Antioxidant	[54]
26.406	Methyl palmitate	Ester	Antioxidant, flavor, nematicide, hypocholesterolermic	
26.716	3-Deoxy-d-mannoic lactone	Ester	N/A	
26.878	d-Mannose	Sugar moiety	Anti-microbial	[55]
27.059	3-O-Methylhexose	Sugar moiety	N/A	
27.535	Palmitic acid	Carboxylic acid	Anti-inflammatory	[22]
30.273	13-Octadecenoic acid, methyl ester	Ester	N/A	
30.621	Methyl linoleate	Ester	N/A	
31.678	Oleic Acid	Carboxylic acid	Anti-inflammatory	[23]
32.111	Linoleic acid	Carboxylic acid	Anti-hyperlipidemic	[24]
32.840	Linolenic acid	Carboxylic acid	Anti-inflammatory	[56]
34.197	Fumaric acid, decyl 2-dimethylaminoethyl ester	Ester	N/A	
34.240	Octanoic acid, 2-dimethylaminoethyl ester	Ester	N/A	
36.021	Glycidyl palmitate	Ester	N/A	
36.635	Palmidrol	Amide	Anti-inflammatory	[57]
37.064	4,5-Dihydro-2-[(8Z,11Z)-8,11 heptadecadienyl]oxazole	Heterocyclic compound	N/A	
39.192	3-Cyclopentylpropionic acid, 2-dimethylaminoethyl ester	Ester	N/A	
39.211	Fumaric acid, 2-dimethylaminoethyl nonyl ester	Ester	N/A	
40.759	2-Phenyl-1,3-dioxan-5-yl 9,12,15-octadecatrienoate	Ester	N/A	
41.611	2-Monopalmitin	Ester	N/A	
44.068	Squalene	Triterpene	Antioxidant	[58]
44.402	Monoolein	Ester	Absorption enhancer	[59]
44.745	β-Monolinolein	Ester	N/A	
46.754	δ-Tocopherol	Tocopherol	Anti-inflammatory, antioxidant	[60]
47.811	γ-Tocopherol	Tocopherol	Anti-inflammatory, antioxidant	[60]
47.911	Stigmastan-3,5-diene	Phytosterol	N/A	
48.597	α-Tocopherol	Tocopherol	Anti-inflammatory, antioxidant	[60]
50.430	Campesterol	Phytosterol	Antioxidant	[61]
50.773	Stigmasterol	Phytosterol	Anti-hyperglycemic, antioxidant	[62]
51.626	γ-Sitosterol	Phytosterol	Anti-hyperglycemic	[63]

Phenolic compounds have been reported to be significantly associated with anti-microbial and antioxidant activities [16, 17]. Two phenolic compounds, 4-vinylguaiacol and 2,3-dimethoxyphenol, were identified. 4-Vinylguaiacol was found in three genotypes possessing soybean rust resistance gene: PI 417132 (*Rpp*3), PI 200487 and PI 200526 (*Rpp*5). Regarding another phenolic compound 2,3-dimethoxyphenol, it was detected in 19 soybean genotypes, which include 11 lines containing soybean rust resistance gene (either *Rpp*1, or *Rpp*3, *Rpp*5, *Rpp*6) and three lines susceptible to soybean rust, two lines resistant to Phomopsis seed decay and one line susceptible to Phomopsis seed decay (S1 Table). How soybean genotypes with different levels of resistance or susceptibility related to the presence of those compounds is uncertain. It is worth noting that no phenolic compounds were identified from soybean genotype PI 518671 (Williams 82), which is a well-known susceptible genotype to soybean rust [18], Phomopsis seed decay [19, 20], and purple seed stain [21].

Five carboxylic acids were identified in total. Notably, palmitic acid and oleic acid, both recognized for their anti-inflammatory properties [22, 23], were detected in all genotypes. Linoleic acid, which is known to possess anti-hyperlipidemic effects [24] was also detected in all samples. Another compound, linolenic acid, which has been reported to have anti-inflammatory activity, was absent in samples SBR3-SBR6 (PI 594538A, PI 594538B, PI 230970, PI 567025A) and SBR9-SBR10 (PI 605854B and PI 605891A). A PSD-resistant genotype, SBR50 (PI 424324B) had nearly four times the amount (6.71 mg/g) of stigmasterol in dried soybean sample as SBR46 (PI 51867), which had 3.06 mg/g of stigmasterol. This soybean line was susceptible to SBR, PSD, and PSS in our previous tests [18–21]. Another PSD resistant genotype, SBR50 (PI 549020), had 3.86 mg/g of stigmasterol. One unknown genotype, SBR41 (PI 471208), had the highest content of stigmasterol. Further study is needed to test if soybean line PI 471208 is resistant to PSD.

### Chemometrics analysis using GC-MS data

**Fig 3:**
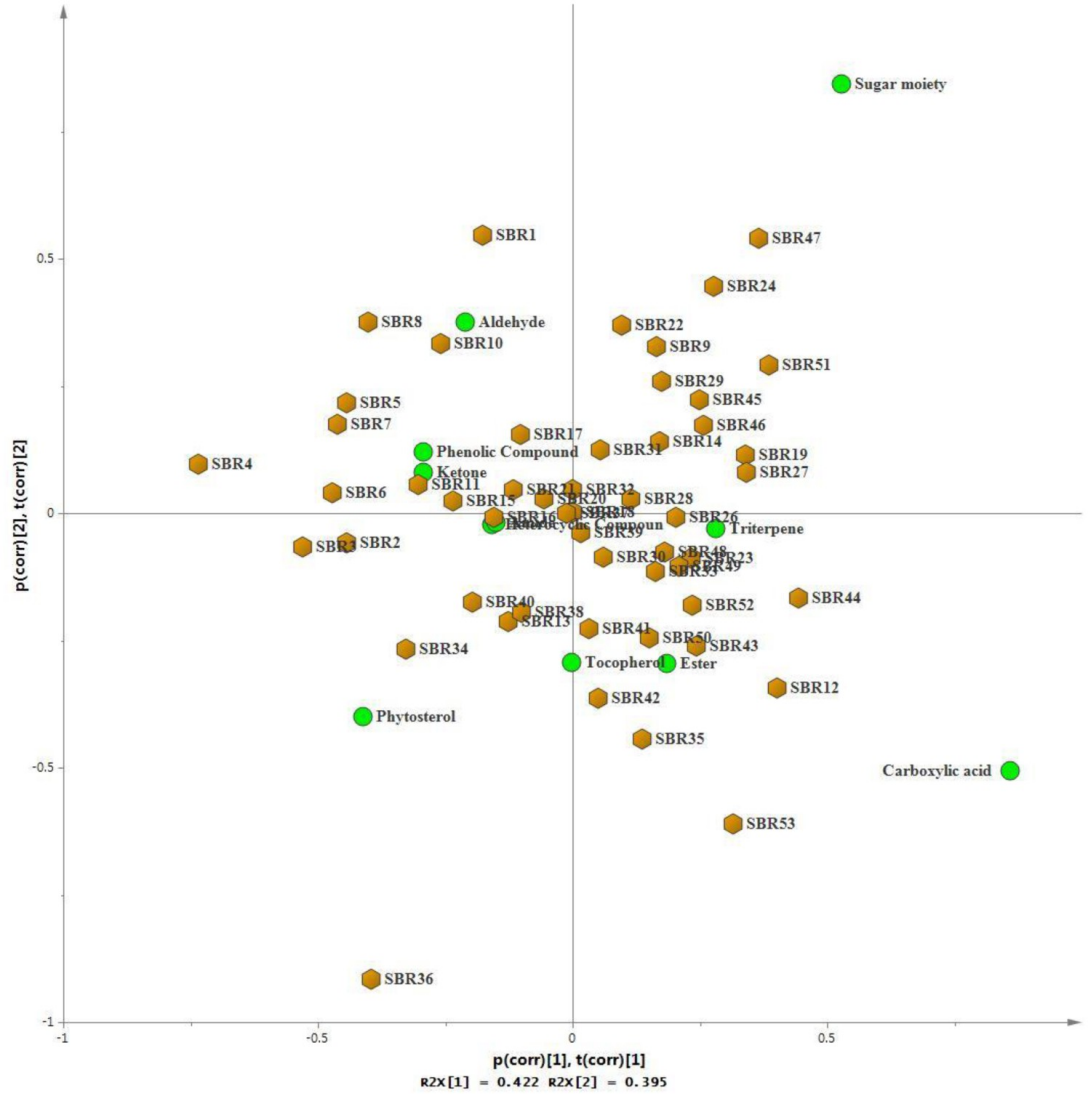
Biplot of 52 soybean genotypes based on 11 phytochemical classes. SBR #1-#36, #45, #50–51, and #53 were resistant soybean accessions; SBR #46-#49, and #52 were susceptible soybean accessions; and SBR #37-#44 were unknown.

In S1 Table, compounds present in various soybean samples were identified. Utilizing this information, we classified each compound into its respective group and aggregated them to produce S2 Table of Supplementary Material for principal component analysis (PCA). Findings from PCA revealed three principal components (PCs) as shown in Table 3 and Fig 3. These components collectively accounted for 89.87% of the total variance observed among the genotypes. For each main component, greater positive coefficients represented a significant factor. PC1 indicated 42.23% of total variance and was mainly composed of the phytochemical classes, carboxylic acid, sugar moiety, and ester. These classes exhibit a significant positive loading on PC1. Genotypes SBR53 (PI 549020), SBR12 (PI 417503), SBR47 (PI 556625), SBR24 (PI 506764), SBR43 (PI 567351B), and SBR51 (PI 458130) showed the most variability according to these components. PC2 illustrated 39.54% of the total variance and classes with higher scores were sugar moiety, aldehyde, and ketone. The genotypes that showed most variance based on this component were SBR47 (PI556625), SBR24 (PI 506764), SBR1 (PI 587880A), SBR8 (PI 567039), SBR10 (PI 605891A, and SBR11 (PI 605865B). PC3 accounted for 8.11% of the total variance, with ester, tocopherol, and heterocyclic compound exhibiting higher scores. Among genotypes, SBR42 (PI 606440A), SBR43 (PI 567351B), SBR16 (PI 606405), and SBR41 (PI 471208) displayed the greatest variability along this component. Based on these findings, it can be inferred that the most divergent genotypes were SBR24 (PI 506764), SBR47 (PI556625), and SBR43 (PI 567351B), as they exhibited positive loading in at least two out of the three components. The effective utilization of PCA minimized the number of variables required for cultivar classification, thereby enabling soybean researchers to establish more meaningful relationships between key soybean characteristics.

**Table 3:** Eigen values and proportion of the variance explained for the three principal components of the 52 soybean genotypes based on phytochemical components.

	PC1	PC2	PC3
**Eigen value**	4.65	4.35	0.89
**Percentage of variance**	42.23	39.54	8.11
**Cumulative percentage**	42.23	81.77	89.89
**Ester**	0.08	-0.14	0.89
**Ketone**	-0.06	0.02	-0.05
**Heterocyclic compound**	-0.01	0.00	0.03
**Carboxylic acid**	0.84	-0.51	-0.16
**Aldehyde**	-0.05	0.09	-0.07
**Phenolic compound**	-0.02	0.01	0.01
**Sugar moiety**	0.50	0.82	-0.03
**Amide**	0.00	0.00	0.02
**Triterpene**	0.01	0.00	0.00
**Tocopherol**	0.00	-0.04	0.05
**Phytosterol**	-0.19	-0.19	-0.40

### UHPLC/DAD-MC analysis of isoflavone components

**Fig 4:**
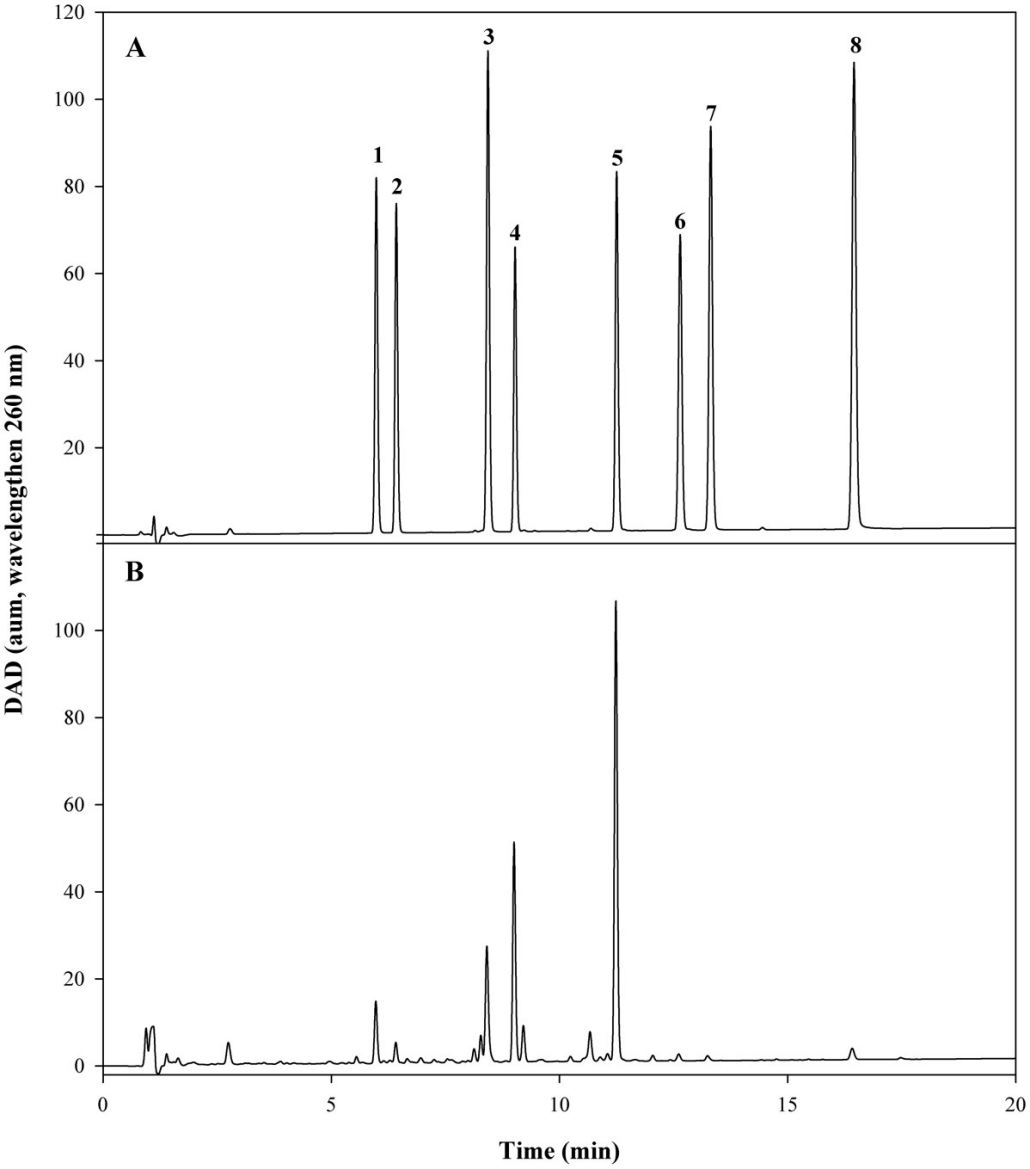
UHPLC/DAD (260 nm) chromatograms of (A) reference standards; (B) soybean seed sample (PI230970, SBR5). Peak identification: 1. daidzin; 2. glycitin; 3. genistin; 4. malonyldaidzin; 5. malonylglycitin; 6. daidzein; 7. glycitein; 8. genistein.

Five major isoflavone components, viz. daidzin (**1**), glycitin (**2**), genistin (**3**), malonyldaidzin (**4**), and malonylglycitin (**5**), were identified and quantified in this study. The structures of these compounds are shown in Fig 1. Chromatograms of the eight isoflavone reference standards and seed samples from different soybean genotypes are illustrated in Fig 4. Quantification results for each isoflavone component can be found in Table 4. While there is variability in the isoflavone content among soybean genotypes, the top five highest concentrations of daidzin, glycitin, genistin, and malonyldaidzin were observed in all three soybean genotypes containing the soybean rust *Rpp6* gene (PI 567102B, PI 567104B, PI 567129). In previous analyses of soybean leaves, an increase accumulation of the isoflavonoids genistein and daidzein occurred in leaves after inoculation with the soybean rust causal pathogen *P*. *pachyrhizi* [28]. Those two isoflavones could be the key phytochemicals contributing to the resistant responses of soybean lines with the *Rpp6* resistant gene. In our study, PI 230970 (*Rpp2*) exhibited the highest level of genistin. However, no genistin was detected in another reported soybean rust resistant genotype SBR 45 (PI 417125) possessing *Rpp2* resistant gene. Pathogenicity experiments are needed to perform to determine if SBR 45 possess the *Rpp2* resistant reaction after inoculating with *P*. *pachyrhizi*. In addition, SBR 29 (PI 200456), which carries the soybean rust *Rpp5* resistance gene, contained 0.09 (mg/g) of glycitein, a compound that was absent in all other 51 tested genotypes including five other genotypes with *Rpp 5* gene (Table 1). The role of glycitein in the resistant response of the soybean PI 200456, however, is not known.

**Table 4:** Isoflavone content in 70% ethanolic seed extract of 52 soybean genotypes analyzed using HPLC/DAD-MS.

Sample Code	Accession (PI)	Daidzin (mg/g)	Glycitin (mg/g)	Genistin (mg/g)	Malonyldaidzin (mg/g)	Malonylgenistin (mg/g)	Daidzein (mg/g)	Glycitein (mg/g)	Genistein (mg/g)
SBR1	587880A	nd	0.06	nd	0.19	0.26	nd^a^	nd	nd
SBR2	200492	0.02	nd	nd	0.46	0.65	nd	nd	nd
SBR3	594538A	nd	nd	nd	0.14	0.34	nd	nd	nd
SBR4	594538B	0.19	0.07	0.08	1.11	1.01	nd	nd	nd
SBR5	230970	0.2	0.06	0.23	1.04	1.67	nd	nd	nd
SBR6	567025A	0.05	0.11	0.05	0.48	0.68	nd	nd	nd
SBR7	567046A	0.14	0.04	0.06	0.91	0.89	nd	nd	nd
SBR8	567039	0.03	0.05	0.02	0.61	0.84	nd	nd	nd
SBR9	605854B	nd	0.1	nd	0.33	0.48	nd	nd	nd
SBR10	605891A	nd	0.07	nd	0.22	0.3	nd	nd	nd
SBR11	605865B	0.01	0.09	0.04	0.38	0.6	nd	nd	nd
SBR12	417503	nd	0.01	nd	0.25	0.29	nd	nd	nd
SBR13	416826A	nd	nd	0.06	0.16	0.49	nd	nd	nd
SBR14	417132	nd	nd	nd	0.24	0.25	nd	nd	nd
SBR15	605885B	0.01	0.03	nd	0.37	0.6	nd	nd	nd
SBR16	606405	nd	0.05	0.01	0.22	0.44	nd	nd	nd
SBR17	605838	nd	0.05	nd	0.19	0.33	nd	nd	nd
SBR18	615437	nd	0.01	nd	0.28	0.44	nd	nd	nd
SBR19	417089A	0.02	nd	nd	0.31	0.21	nd	nd	nd
SBR20	567056B	0.07	0.06	0.02	0.66	0.65	nd	nd	nd
SBR21	462312	0.1	nd	0.11	1.06	1.49	nd	nd	nd
SBR22	567099A	0.25	nd	0.08	0.91	0.58	nd	nd	nd
SBR23	635999	nd	nd	nd	0.27	0.38	nd	nd	nd
SBR24	506764	nd	nd	nd	0.16	0.13	nd	nd	nd
SBR26	417089B	nd	nd	nd	0.25	0.21	nd	nd	nd
SBR27	605791A	0.08	nd	0.09	0.74	1.16	nd	nd	nd
SBR28	459025B	nd	nd	nd	0.03	0.05	nd	nd	nd
SBR29	200456	nd	nd	nd	0.11	0.16	nd	0.09	nd
SBR30	200487	0.01	nd	nd	0.52	0.71	nd	nd	nd
SBR31	471904	nd	0.09	0.04	0.3	0.62	nd	nd	nd
SBR32	200526	nd	nd	nd	0.31	0.34	nd	nd	nd
SBR33	567129	0.25	0.11	0.12	1.16	0.92	nd	nd	nd
SBR34	567104B	0.45	0.11	0.19	1.76	1.16	nd	nd	nd
SBR35	567102B	0.37	0.12	0.19	1.59	1.23	nd	nd	nd
SBR36	605823	nd	nd	nd	0.11	0.16	nd	nd	nd
SBR37	605773	nd	nd	nd	0.44	0.41	nd	nd	nd
SBR38	567189A	0.04	0.05	0.05	0.67	1.14	nd	nd	nd
SBR39	224268	0.05	nd	0.04	0.61	0.87	nd	nd	nd
SBR40	203398	0.13	nd	0.08	1.12	1.16	nd	nd	nd
SBR41	471208	0.03	nd	nd	0.52	0.65	nd	nd	nd
SBR42	606440A	0.13	0.02	0.13	0.98	1.40	nd	nd	nd
SBR43	567351B	0.09	0.04	0.11	1.10	1.41	nd	nd	nd
SBR44	470227B	0.02	nd	0.03	0.56	0.89	nd	nd	nd
SBR45	417125	nd	nd	nd	0.41	0.58	nd	nd	nd
SBR46	518671	0.10	0.01	0.08	0.89	1.27	nd	nd	nd
SBR47	556625	0.03	0.01	nd	0.44	0.47	nd	nd	nd
SBR48	587886	nd	nd	nd	0.2	0.27	nd	nd	nd
SBR49	605833	nd	0.03	nd	0.21	0.2	nd	nd	nd
SBR50	424324B	nd	nd	nd	0.2	0.59	nd	nd	nd
SBR51	458130	nd	nd	nd	0.17	0.21	nd	nd	nd
SBR52	399045	0.07	nd	nd	0.54	0.58	nd	nd	nd
SBR53	549020	nd	nd	nd	0.21	0.19	nd	nd	nd

^a^: Not detected. Resistant soybean accessions were highlighted in green, susceptible accessions were highlighted in yellow; unhighlighted accessions were unknown.

Isoflavones are a group of phenolic compounds commonly found in the legume (Fabaceae) family. Soybean isoflavone is an important secondary metabolite accumulated in soybean. It has been reported that isoflavones contribute to overall human health, including chronic diseases [25, 26]. Isoflavones have also been reported to mediate important interactions with plant-associated microbes, including defense from pathogens and in nodulation [27].

Soybean rust (SBR) is one of the most important soybean foliar diseases occurring in many major soybean-producing countries. In a reported study on the importance of phenolic metabolism to limit the growth of *P*. *pachyrhizi* that causes soybean rust, it was found that inoculation of soybean plants with the pathogen resulted in increased accumulation of isoflavonoids and flavonoids in leaves of all soybean genotypes tested [28]. Although the soybean phytoalexin glyceollin was not detected in leaves of uninfected plants, accumulation of this compound at marked levels occurred in rust-infected leaves [28]. In another study testing a susceptible genotype PI 636463, significant production of defense secondary metabolites including phenylpropanoids, terpenoids and flavonoids were found when *P*. *pachyrhizi* infected soybean [29]. Exploration of the relationship between isoflavone content and soybean genotypes with known resistance genes would help reveal the biochemical basis of soybean resistance to disease. Experiments are underway to analyze isoflavone content of soybean resistant and susceptible genotypes after pathogen (*P*. *pachyrhizi* and *D*. *longicolla*) inoculation and non-inoculation treatments.

In this study, a conclusion could not be drawn about the direct correlations between the studied compounds and phenotypic traits illustrating the resistance/susceptibility to the pathogens. To address this issue, phytopathogenic experiments will be conducted to test selected representative genotypes, such as PI 518671 (Williams 82), which is a well-known susceptible genotype to soybean rust [18], Phomopsis seed decay [19, 20], and purple seed stain [21], as well as the resistant line PI 567102B that contains the soybean rust *Rpp6* gene [18]. Soybean samples will be collected from replicated tests directly for the phytochemical analyses.

Together, information obtained from comprehensive phytochemical profiling of soybean genotypes with their reactions to pathogens will facilitate selection of soybean lines for breeding for resistance to diseases.

## Conclusion

In this study, a comprehensive phytochemical profiling of soybean genotypes was conducted to explore various reactions to soybean diseases including soybean rust, Phomopsis seed decay, and purple seed stain using GC-MS and UHPLC/DAD-MS. The results revealed significant diversity in the isoflavone profiles among the genotypes studied. Notably, certain genotypes containing specific soybean rust resistance genes exhibited higher levels of particular isoflavone components compared to others. For example, genotypes carrying the soybean rust *Rpp6* gene consistently contained elevated levels of daidzin, glycitin, genistin, and malonyldaidzin. Furthermore, genotypes harboring the *Rpp2* (SBR5) gene had a distinct accumulation of genistin and malonyldaidzin, while one genotype possessing the *Rpp5* gene (SBR29) exclusively contained the compound glycitein. These results highlight the potential correlation between soybean rust resistance genes and the biosynthesis of key phytochemicals in soybean, underscoring the importance of genetic factors in shaping the phytochemical composition of soybean varieties. Such insights could contribute to the development of disease-resistant soybean cultivars with enhanced nutritional and functional attributes. Overall, this study sheds light on the intricate interplay between genetics, phytochemistry, and disease resistance in soybeans, offering valuable implications for soybean breeding and agricultural practices.

## Supporting information

S1 Table List of important phytocompounds tentatively identified in the methanolic seed extract of soybean genotypes by GC-MS. (DOCX)

S2 Table Compositional data expressed as different groups of compounds used for PCA. (DOCX)
