# Supplementary material for: Phytochemical profiling of soybean genotypes using GC-MS and UHPLC-DAD/MS
Source: PLoS One. 2024 Aug 15;19(8):e0308489. doi: 10.1371/journal.pone.0308489 (PMC11326653; doi:10.1371/journal.pone.0308489)
Supplement: S1 Table — (DOCX) [file pone.0308489.s001.docx]

**Supporting Information (S1 Table)**

**Phytochemical profiling of soybean genotypes with different reactions to soybean diseases using GC-MS and UHPLC/DAD-MS**

Shuxian Li^1*^, Mei Wang^2^, Joseph Lee^3^

**^1^** United States Department of Agriculture, Agricultural Research Service (USDA, ARS), Crop Genetics Research Unit, Stoneville, MS 38776, USA.

**^2^** USDA, ARS, Natural Products Utilization Research Unit, University, MS 38677, USA.

**^3^** National Center for Natural Products Research, School of Pharmacy, University of Mississippi, University, MS 38677, USA.

**S1 Table.** List of important phytocompounds tentatively identified in the methanolic seed extract of soybean genotypes and their % peak area by GC-MS.^a^

| ***R_t_*** | **Compound Identification** | **SBR1** | **SBR2** | **SBR3** | **SBR4** | **SBR5** | **SBR6** | **SBR7** | **SBR8** | **SBR9** | **SBR10** | **SBR11** | **SBR12** | **SBR13** |
| --- | --- | --- | --- | --- | --- | --- | --- | --- | --- | --- | --- | --- | --- | --- |
| 5.044 | Methyl pyruvate | 0.84 | 0.98 | 0.88 | 0.71 | 0.65 | 0.68 | 0.79 | 0.63 | 0.94 | 0.61 | 0.68 | 0.70 | 0.65 |
| 7.763 | Dihydroxyacetone | 0.28 | 0.13 | 0.28 | 0.57 | 0.32 | 0.35 | 0.38 | 0.72 | nd | nd | 0.29 | nd | 0.05 |
| 8.068 | 2,4-Dihydroxy-2,5-dimethyl-3(2H)-furan-3-one | 0.38 | 0.47 | 0.81 | 0.80 | 0.87 | 0.70 | 0.99 | 1.30 | 1.12 | 0.70 | 0.77 | nd | 0.94 |
| 8.254 | Pyranone | nd | nd | nd | nd | nd | nd | nd | nd | nd | nd | nd | nd | nd |
| 11.530 | 2-Hydroxy-γ-butyrolactone | 0.62 | 0.46 | 0.47 | 0.44 | 0.66 | 0.29 | 0.47 | 0.32 | nd | 0.29 | 0.53 | nd | 0.32 |
| 11.887 | Maltol | 0.68 | 0.59 | 1.16 | 1.16 | 1.50 | 1.51 | 1.81 | 2.20 | 2.03 | 1.25 | 1.40 | nd | 1.59 |
| 12.025 | l-Alanine, n-propargyloxycarbonyl-, hexyl ester | 0.11 | 0.29 | nd | nd | nd | nd | 0.07 | nd | nd | nd | nd | nd | nd |
| 12.168 | 4,5-Diamino-2-hydroxypyrimidine | nd | nd | nd | nd | nd | nd | nd | nd | nd | nd | nd | nd | nd |
| 15.101 | Serine, acetate | 0.27 | 0.28 | 0.32 | 0.46 | 0.39 | 0.56 | nd | 0.53 | 1.03 | nd | nd | nd | nd |
| 15.130 | l-Alanine, N-methoxycarbonyl-, heptyl ester | nd | nd | nd | nd | nd | nd | 0.47 | nd | nd | 0.26 | 0.72 | nd | 0.78 |
| 15.144 | α-Methylacetoacetic ester | nd | nd | nd | nd | nd | nd | nd | nd | nd | nd | nd | nd | nd |
| 15.316 | Propanoic acid, 3-(acetylthio)-2-methyl- | nd | nd | nd | nd | nd | nd | nd | nd | nd | nd | nd | nd | nd |
| 17.211 | 5-Hydroxymethylfurfural | 2.81 | 2.10 | 2.52 | 4.47 | 3.84 | 3.70 | 5.10 | 6.14 | 4.84 | 2.40 | 3.58 | 2.77 | 3.09 |
| 17.625 | 4-Vinylguaiacol | 0.20 | 0.32 | 0.28 | 0.22 | 0.45 | 0.28 | 0.15 | 0.13 | nd | 0.14 | 0.14 | nd | 0.25 |
| 19.440 | 2,3-Dimethoxyphenol | 1.04 | 1.02 | 0.47 | 0.47 | 0.62 | 0.44 | 0.42 | 0.46 | nd | 0.53 | 0.67 | 0.89 | 0.46 |
| 22.916 | Sucrose | 21.64 | 16.98 | 16.98 | 19.59 | 20.32 | 20.06 | 22.28 | 25.34 | 28.66 | 17.45 | 20.04 | 12.16 | 18.49 |
| 23.759 | 3,5-Dimethoxyacetophenone | 0.95 | 1.05 | 0.51 | 1.01 | 1.27 | 1.04 | 1.23 | 1.22 | 1.90 | 0.99 | 1.18 | 0.12 | 0.65 |
| 26.406 | Methyl palmitate | 0.42 | 0.31 | nd | nd | nd | nd | 0.64 | 0.73 | 0.86 | 0.48 | 0.42 | nd | 0.81 |
| 26.716 | 3-Deoxy-d-mannoic lactone | nd | nd | nd | 1.17 | nd | nd | 1.01 | 0.44 | nd | nd | nd | nd | nd |
| 26.878 | d-Mannose | nd | nd | nd | 0.42 | nd | 1.33 | nd | nd | nd | nd | nd | nd | nd |
| 27.059 | 3-O-Methylhexose | 10.75 | 2.71 | 2.84 | nd | 3.73 | nd | 1.27 | 2.23 | 5.56 | 10.96 | 3.54 | 11.79 | 1.87 |
| 27.535 | Palmitic acid | 4.03 | 5.45 | 5.25 | 3.90 | 4.78 | 7.12 | 5.14 | 4.33 | 7.83 | 5.81 | 6.27 | 11.03 | 8.07 |
| 30.273 | 13-Octadecenoic acid, methyl ester | 0.08 | 0.14 | 15.00 | nd | nd | 0.10 | 0.12 | 0.13 | nd | 0.26 | 0.18 | nd | 0.17 |
| 30.621 | Methyl linoleate | 0.18 | 0.31 | nd | 0.22 | 0.30 | 0.40 | 0.38 | 0.32 | 0.92 | 0.67 | 0.64 | 0.34 | 0.72 |
| 31.678 | Oleic acid | 4.73 | 3.69 | 2.59 | 1.91 | 1.84 | 1.84 | 2.61 | 3.03 | 4.48 | 3.53 | 3.79 | 5.77 | 3.08 |
| 32.111 | Linoleic acid | 8.64 | 10.85 | 9.04 | 8.42 | 10.92 | 9.86 | 9.82 | 8.95 | 15.44 | 10.27 | 11.19 | 19.86 | 15.20 |
| 32.840 | Linolenic acid | 0.63 | 0.16 | nd | nd | nd | nd | 0.36 | 0.45 | nd | nd | 0.40 | 0.86 | 0.75 |
| 34.197 | Fumaric acid, decyl 2-dimethylaminoethyl ester | 0.61 | 1.33 | 0.67 | 0.75 | 0.71 | 0.83 | 0.82 | 0.57 | nd | 0.75 | 0.71 | 0.89 | 0.68 |
| 34.240 | Octanoic acid, 2-dimethylaminoethyl ester | nd | 0.81 | nd | nd | nd | nd | nd | nd | nd | nd | nd | 0.97 | nd |
| 36.021 | Glycidyl palmitate | nd | 0.50 | 0.46 | 0.41 | 0.40 | 0.46 | 0.41 | 0.38 | nd | 0.41 | 0.39 | 0.38 | 0.41 |
| 36.635 | Palmidrol | 0.38 | 0.34 | 0.75 | 0.33 | 0.31 | 0.34 | nd | 0.55 | nd | 0.53 | 0.37 | 0.29 | 0.62 |
| 37.064 | 4,5-Dihydro-2-[(8Z,11Z)-8,11-heptadecadienyl]oxazole | 1.46 | 1.01 | 2.75 | 1.42 | 1.93 | 1.66 | 1.40 | 1.47 | 2.00 | 1.80 | 1.24 | 1.48 | 3.23 |
| 39.192 | 3-Cyclopentylpropionic acid, 2-dimethylaminoethyl ester | 2.93 | 2.47 | 1.85 | 2.54 | 2.25 | 2.58 | 2.25 | nd | nd | 0.54 | 1.77 | 2.72 | 1.78 |
| 39.211 | Fumaric acid, 2-dimethylaminoethyl nonyl ester | 2.09 | nd | nd | nd | nd | nd | nd | 1.77 | nd | 1.85 | nd | nd | nd |
| 40.759 | 2-Phenyl-1,3-dioxan-5-yl 9,12,15-octadecatrienoate | 1.02 | 1.16 | 0.89 | 0.90 | 0.86 | 0.99 | 0.89 | 0.91 | 1.20 | 0.86 | 0.71 | 0.81 | 0.72 |
| 41.611 | 2-Monopalmitin | 0.13 | 0.45 | nd | 0.09 | 0.43 | 1.51 | 0.45 | 1.29 | 1.81 | 0.37 | 0.97 | 2.53 | 2.07 |
| 44.068 | Squalene | nd | nd | nd | nd | nd | nd | nd | nd | nd | nd | nd | nd | nd |
| 44.402 | Monoolein | nd | nd | nd | nd | nd | nd | nd | nd | nd | nd | nd | 0.31 | nd |
| 44.745 | β-Monolinolein | 1.70 | nd | 2.27 | nd | 1.96 | 1.48 | nd | 1.90 | nd | nd | 1.91 | 1.50 | 2.11 |
| 46.754 | Δ-Tocopherol | 0.89 | 0.65 | 1.76 | 1.40 | 1.09 | 1.27 | 0.59 | 1.36 | 1.03 | 0.77 | 0.77 | 0.66 | 1.24 |
| 47.811 | γ-Tocopherol | 2.95 | 2.37 | 3.22 | 1.99 | 2.24 | 2.89 | 1.48 | 1.81 | 3.89 | 2.62 | 2.86 | 2.58 | 3.04 |
| 47.911 | Stigmastan-3,5-diene | nd | nd | nd | nd | nd | nd | nd | nd | nd | nd | nd | 0.49 | nd |
| 48.597 | α-Tocopherol | 0.43 | nd | nd | nd | nd | nd | nd | nd | nd | nd | nd | 0.67 | 0.75 |
| 50.430 | Campesterol | 1.20 | 1.83 | 2.39 | 3.77 | 1.77 | 1.89 | 1.54 | 1.68 | 2.96 | 2.00 | 2.41 | 2.07 | 1.76 |
| 50.773 | Stigmasterol | 1.69 | 3.32 | 4.34 | 6.07 | 2.18 | 3.42 | 4.65 | 2.78 | 4.44 | 3.05 | 3.27 | 2.82 | 3.09 |
| 51.626 | γ-Sitosterol | 4.59 | 6.03 | 4.87 | 4.95 | 5.74 | 6.94 | 5.61 | 6.33 | 9.07 | 5.99 | 7.00 | 6.44 | 5.72 |

**S1 Table.** Continue...

| ***R_t_*** | **Compound Identification** | **SBR14** | **SBR15** | **SBR16** | **SBR17** | **SBR18** | **SBR19** | **SBR20** | **SBR21** | **SBR22** | **SBR23** | **SBR24** | **SBR26** | **SBR27** |
| --- | --- | --- | --- | --- | --- | --- | --- | --- | --- | --- | --- | --- | --- | --- |
| 5.044 | Methyl pyruvate | 0.62 | 0.64 | 0.71 | 0.77 | 0.45 | 0.79 | 0.63 | 0.70 | 1.10 | 0.75 | 0.01 | 0.42 | 0.81 |
| 7.763 | Dihydroxyacetone | 0.12 | nd | 0.27 | 0.10 | nd | 0.18 | nd | nd | 0.18 | 0.01 | nd | nd | nd |
| 8.068 | 2,4-Dihydroxy-2,5-dimethyl-3(2H)-furan-3-one | 0.44 | 0.51 | 0.69 | 0.71 | 0.48 | 0.49 | 0.77 | 0.92 | 0.50 | 0.77 | 0.63 | 0.67 | 0.81 |
| 8.254 | Pyranone | nd | nd | nd | nd | nd | nd | nd | nd | nd | nd | nd | nd | nd |
| 11.530 | 2-Hydroxy-γ-butyrolactone | 0.18 | 0.31 | 0.29 | 0.34 | 0.40 | 0.23 | nd | 0.44 | 0.21 | 0.16 | 0.91 | 0.60 | 1.35 |
| 11.887 | Maltol | 1.10 | 1.03 | 1.13 | 1.09 | 0.82 | 1.69 | 0.16 | 1.14 | 1.20 | 0.64 | 0.95 | 1.43 | 1.33 |
| 12.025 | l-Alanine, n-propargyloxycarbonyl-, hexyl ester | nd | nd | nd | nd | nd | nd | 0.21 | nd | nd | nd | nd | nd | nd |
| 12.168 | 4,5-Diamino-2-hydroxypyrimidine | nd | nd | nd | nd | nd | nd | nd | nd | nd | nd | nd | nd | nd |
| 15.101 | Serine, acetate | nd | nd | nd | nd | nd | nd | nd | 1.04 | nd | nd | nd | nd | nd |
| 15.130 | l-Alanine, N-methoxycarbonyl-, heptyl ester | 0.27 | nd | nd | nd | 0.29 | 0.17 | 0.21 | nd | nd | nd | 0.19 | nd | nd |
| 15.144 | α-Methylacetoacetic ester | nd | 0.37 | 0.69 | 0.72 | nd | nd | nd | nd | 0.73 | nd | nd | nd | nd |
| 15.316 | Propanoic acid, 3-(acetylthio)-2-methyl- | nd | nd | nd | nd | nd | nd | nd | nd | nd | nd | nd | nd | nd |
| 17.211 | 5-Hydroxymethylfurfural | 2.37 | 2.34 | 3.65 | 3.92 | 1.88 | 1.83 | 1.23 | 3.50 | 3.43 | 1.21 | 2.18 | 5.55 | 1.55 |
| 17.625 | 4-Vinylguaiacol | 0.64 | 0.19 | 0.14 | 0.15 | 0.22 | 0.08 | 0.11 | 0.12 | 0.24 | 0.10 | 0.11 | nd | 0.20 |
| 19.440 | 2,3-Dimethoxyphenol | 0.42 | 0.50 | 0.43 | 0.45 | 0.51 | 0.24 | 0.64 | 0.45 | 0.59 | 0.35 | 0.34 | 0.14 | 0.38 |
| 22.916 | Sucrose | 14.05 | 18.40 | 20.37 | 21.46 | 17.99 | 15.52 | 18.19 | 23.05 | 28.73 | 16.33 | 17.59 | 18.49 | 24.37 |
| 23.759 | 3,5-Dimethoxyacetophenone | 0.66 | 0.76 | 0.92 | 0.72 | 0.77 | 0.60 | 1.16 | 0.99 | 1.17 | 0.78 | 0.63 | 0.09 | 0.86 |
| 26.406 | Methyl palmitate | 0.28 | 0.34 | 0.31 | 0.56 | 0.69 | 0.47 | 0.80 | 0.80 | 0.94 | 0.92 | 0.65 | 0.66 | 1.00 |
| 26.716 | 3-Deoxy-d-mannoic lactone | nd | nd | nd | nd | nd | nd | nd | nd | nd | nd | nd | nd | nd |
| 26.878 | d-Mannose | nd | nd | nd | nd | nd | nd | nd | nd | nd | nd | nd | nd | nd |
| 27.059 | 3-O-Methylhexose | 14.98 | 5.79 | 4.01 | 5.76 | 7.92 | 14.44 | 7.91 | 2.64 | 4.20 | 9.92 | 17.84 | 7.99 | 5.62 |
| 27.535 | Palmitic acid | 7.94 | 6.61 | 6.58 | 6.30 | 7.09 | 7.42 | 7.01 | 6.18 | 8.90 | 8.05 | 6.52 | 8.35 | 8.35 |
| 30.273 | 13-Octadecenoic acid, methyl ester | 0.19 | 0.24 | 0.42 | 0.34 | 0.37 | 0.32 | 0.35 | 0.28 | 0.25 | 0.94 | 0.30 | 0.28 | 0.40 |
| 30.621 | Methyl linoleate | 0.82 | 0.81 | 0.75 | 0.71 | 1.25 | 0.70 | 1.05 | 1.35 | 0.70 | 1.66 | 1.34 | 0.93 | 1.41 |
| 31.678 | Oleic acid | 3.51 | 3.80 | 6.45 | 6.12 | 4.32 | 7.38 | 4.18 | 2.08 | 4.16 | 7.39 | 3.48 | 4.39 | 4.61 |
| 32.111 | Linoleic acid | 16.36 | 11.81 | 10.96 | 11.10 | 14.88 | 15.95 | 13.59 | 14.91 | 11.44 | 15.66 | 15.98 | 17.03 | 17.99 |
| 32.840 | Linolenic acid | 0.45 | 0.58 | 0.46 | 0.35 | 0.55 | 0.86 | 0.94 | 0.90 | 0.66 | 0.58 | 0.64 | 0.85 | 0.78 |
| 34.197 | Fumaric acid, decyl 2-dimethylaminoethyl ester | 0.67 | 0.75 | 0.84 | 0.73 | 0.68 | 0.50 | 0.87 | 0.69 | 0.61 | 0.86 | 0.76 | 0.55 | 0.73 |
| 34.240 | Octanoic acid, 2-dimethylaminoethyl ester | nd | nd | nd | nd | nd | nd | nd | nd | nd | nd | nd | nd | nd |
| 36.021 | Glycidyl palmitate | 0.40 | 0.41 | 0.35 | 0.35 | 0.36 | 0.23 | 0.45 | 0.36 | 0.33 | 0.43 | 0.41 | 0.27 | 0.42 |
| 36.635 | Palmidrol | nd | 0.33 | 0.51 | 0.81 | nd | 0.93 | 0.35 | 0.29 | 0.50 | 0.53 | 0.21 | 0.46 | 0.30 |
| 37.064 | 4,5-Dihydro-2-[(8Z,11Z)-8,11-heptadecadienyl]oxazole | 1.62 | 1.09 | 0.81 | 1.63 | 1.52 | 2.05 | 1.41 | 1.26 | 1.75 | 1.29 | 1.39 | 2.12 | 1.56 |
| 39.192 | 3-Cyclopentylpropionic acid, 2-dimethylaminoethyl ester | 1.98 | 1.84 | 1.01 | 1.79 | 2.02 | 1.35 | 2.58 | 3.58 | 1.51 | 2.25 | 2.15 | 1.37 | 1.57 |
| 39.211 | Fumaric acid, 2-dimethylaminoethyl nonyl ester | nd | nd | 1.91 | nd | nd | nd | nd | nd | nd | nd | nd | nd | nd |
| 40.759 | 2-Phenyl-1,3-dioxan-5-yl 9,12,15-octadecatrienoate | 0.73 | 0.74 | 0.62 | 0.71 | 0.75 | 0.43 | 0.77 | 1.08 | 0.58 | 0.30 | 0.83 | 0.45 | 0.66 |
| 41.611 | 2-Monopalmitin | 2.27 | 2.67 | 2.21 | 2.17 | 2.28 | 1.98 | 1.87 | 1.19 | 1.83 | 1.94 | 1.67 | 2.69 | 1.08 |
| 44.068 | Squalene | nd | nd | nd | nd | nd | nd | nd | nd | 0.76 | nd | 0.18 | 0.27 | 0.13 |
| 44.402 | Monoolein | nd | nd | 0.71 | nd | nd | nd | nd | nd | nd | nd | nd | nd | nd |
| 44.745 | β-Monolinolein | 2.88 | 2.08 | 1.99 | 1.34 | 2.55 | 2.28 | 1.63 | 4.07 | 1.07 | 1.48 | 3.60 | 2.85 | 2.85 |
| 46.754 | Δ-Tocopherol | 0.52 | 1.09 | 0.71 | 0.61 | 0.61 | 0.91 | 0.80 | 1.29 | 0.55 | 0.69 | 0.50 | 0.95 | 0.62 |
| 47.811 | γ-Tocopherol | 2.45 | 3.29 | 2.46 | 2.74 | 2.28 | 3.14 | 2.28 | 2.03 | 1.31 | 2.88 | 2.15 | 3.43 | 1.93 |
| 47.911 | Stigmastan-3,5-diene | nd | nd | nd | nd | nd | nd | nd | 0.30 | 0.17 | 0.16 | 0.16 | 0.28 | 0.18 |
| 48.597 | α-Tocopherol | 0.47 | nd | nd | 0.77 | 0.85 | nd | nd | 0.24 | nd | 0.24 | 0.64 | 0.38 | 0.15 |
| 50.430 | Campesterol | 1.37 | 2.92 | 2.41 | 1.96 | 1.91 | 0.80 | 2.12 | 2.37 | 1.79 | 1.61 | 1.03 | 0.84 | 1.30 |
| 50.773 | Stigmasterol | 1.87 | 3.02 | 2.66 | 3.32 | 4.41 | 1.84 | 4.34 | 2.26 | 1.90 | 2.70 | 1.98 | 2.67 | 1.66 |
| 51.626 | γ-Sitosterol | 4.44 | 7.63 | 8.13 | 6.17 | 5.53 | 3.79 | 6.82 | 7.12 | 7.15 | 5.26 | 3.60 | 3.51 | 4.97 |

**S1 Table.** Continue...

| ***R_t_*** | **Compound Identification** | **SBR28** | **SBR29** | **SBR30** | **SBR31** | **SBR32** | **SBR33** | **SBR34** | **SBR35** | **SBR36** | **SBR37** | **SBR38** | **SBR39** | **SBR40** |
| --- | --- | --- | --- | --- | --- | --- | --- | --- | --- | --- | --- | --- | --- | --- |
| 5.044 | Methyl pyruvate | 0.95 | 0.55 | 0.73 | 0.80 | 0.88 | 0.74 | 0.84 | 0.89 | 0.83 | 0.25 | 0.42 | 0.69 | 0.69 |
| 7.763 | Dihydroxyacetone | nd | 0.08 | nd | 0.06 | nd | nd | 0.07 | nd | nd | nd | nd | nd | nd |
| 8.068 | 2,4-Dihydroxy-2,5-dimethyl-3(2H)-furan-3-one | 0.74 | nd | 1.39 | 0.58 | 0.49 | 0.74 | 0.56 | 1.10 | 2.18 | 2.43 | 3.82 | 1.75 | 2.36 |
| 8.254 | Pyranone | nd | 0.35 | nd | nd | nd | nd | nd | nd | nd | nd | nd | nd | nd |
| 11.530 | 2-Hydroxy-γ-butyrolactone | 0.38 | 0.40 | 0.43 | 0.58 | 0.44 | 0.51 | 0.45 | 0.67 | 0.01 | 0.67 | 0.50 | 0.60 | 0.62 |
| 11.887 | Maltol | 0.31 | 0.85 | 1.66 | 1.21 | 0.45 | 1.62 | 1.03 | 1.03 | 0.02 | 1.90 | 0.99 | 0.84 | 1.80 |
| 12.025 | l-Alanine, n-propargyloxycarbonyl-, hexyl ester | nd | 0.70 | nd | nd | nd | nd | nd | nd | nd | nd | nd | nd | nd |
| 12.168 | 4,5-Diamino-2-hydroxypyrimidine | nd | nd | nd | nd | nd | nd | nd | nd | nd | nd | nd | nd | nd |
| 15.101 | Serine, acetate | nd | nd | nd | nd | nd | 0.17 | nd | nd | nd | nd | 0.22 | nd | nd |
| 15.130 | l-Alanine, N-methoxycarbonyl-, heptyl ester | 0.24 | nd | nd | nd | nd | nd | nd | nd | nd | 0.05 | nd | nd | nd |
| 15.144 | α-Methylacetoacetic ester | nd | nd | 0.28 | nd | nd | nd | nd | nd | nd | nd | nd | nd | nd |
| 15.316 | Propanoic acid, 3-(acetylthio)-2-methyl- | nd | nd | nd | 1.00 | 0.13 | nd | nd | nd | nd | nd | nd | nd | nd |
| 17.211 | 5-Hydroxymethylfurfural | 1.12 | 3.18 | 2.26 | 2.41 | 2.47 | 1.79 | 2.50 | 1.79 | 0.51 | 4.37 | 1.34 | 1.01 | 1.87 |
| 17.625 | 4-Vinylguaiacol | 0.10 | nd | 0.93 | 0.47 | 0.70 | 0.12 | 0.14 | 0.11 | 0.03 | nd | nd | nd | 0.34 |
| 19.440 | 2,3-Dimethoxyphenol | 0.14 | 0.20 | 0.41 | 0.25 | 0.18 | 0.42 | 0.44 | 0.42 | 0.28 | 0.61 | 0.22 | 0.02 | 0.22 |
| 22.916 | Sucrose | 19.59 | 17.80 | 13.37 | 24.19 | 21.63 | 17.56 | 15.61 | 15.03 | 3.07 | 22.20 | 17.25 | 11.26 | 15.68 |
| 23.759 | 3,5-Dimethoxyacetophenone | 0.57 | 0.21 | 0.64 | 0.93 | 0.59 | 1.20 | 0.59 | 0.74 | 0.37 | 0.57 | 0.89 | 0.15 | 0.38 |
| 26.406 | Methyl palmitate | 1.09 | 0.75 | 0.72 | 0.88 | 0.56 | 0.88 | 0.80 | 0.86 | 0.70 | 0.84 | 0.91 | 0.79 | 0.90 |
| 26.716 | 3-Deoxy-d-mannoic lactone | nd | nd | nd | nd | nd | nd | nd | nd | nd | nd | nd | nd | nd |
| 26.878 | d-Mannose | nd | nd | nd | nd | nd | nd | nd | nd | nd | nd | nd | nd | nd |
| 27.059 | 3-O-Methylhexose | 7.64 | 14.19 | 11.59 | 4.41 | 4.37 | 8.37 | 2.99 | 4.31 | 3.77 | 2.37 | 3.85 | 13.36 | 4.04 |
| 27.535 | Palmitic acid | 8.82 | 7.53 | 9.36 | 6.88 | 6.31 | 9.06 | 6.72 | 10.67 | 7.80 | 9.36 | 6.30 | 6.54 | 6.02 |
| 30.273 | 13-Octadecenoic acid, methyl ester | 0.47 | 0.27 | 0.19 | 0.34 | 0.37 | 0.33 | 0.23 | 0.33 | 0.63 | 0.54 | 0.69 | 0.43 | 0.47 |
| 30.621 | Methyl linoleate | 1.67 | 1.43 | 1.13 | 1.32 | 1.48 | 1.03 | 0.80 | 1.06 | 2.82 | 1.89 | 1.66 | 1.81 | 1.98 |
| 31.678 | Oleic acid | 4.12 | 2.37 | 2.32 | 3.89 | 2.51 | 5.32 | 4.70 | 5.75 | 4.28 | 4.01 | 6.17 | 3.95 | 4.40 |
| 32.111 | Linoleic acid | 15.07 | 16.33 | 16.48 | 13.85 | 16.26 | 15.68 | 12.40 | 16.75 | 17.77 | 13.28 | 13.95 | 16.12 | 14.48 |
| 32.840 | Linolenic acid | 0.48 | 0.81 | 0.88 | 0.87 | 0.95 | 1.09 | 0.82 | 0.90 | 0.23 | 0.57 | 0.82 | 0.72 | 0.43 |
| 34.197 | Fumaric acid, decyl 2-dimethylaminoethyl ester | 0.70 | 0.68 | 0.79 | 0.61 | 0.96 | 0.78 | 0.59 | 0.86 | 0.75 | 0.75 | 0.89 | 0.85 | 0.61 |
| 34.240 | Octanoic acid, 2-dimethylaminoethyl ester | nd | nd | nd | nd | nd | nd | nd | nd | nd | nd | nd | nd | nd |
| 36.021 | Glycidyl palmitate | 0.47 | 0.38 | 0.35 | 0.36 | 0.34 | 0.35 | 0.27 | 0.36 | nd | 0.42 | 0.30 | 0.33 | 0.40 |
| 36.635 | Palmidrol | 0.36 | 0.26 | 0.32 | 0.37 | 0.34 | 0.50 | 0.27 | 0.39 | 0.30 | 0.26 | 0.25 | 0.27 | 0.27 |
| 37.064 | 4,5-Dihydro-2-[(8Z,11Z)-8,11-heptadecadienyl]oxazole | 1.61 | 1.69 | 2.60 | 1.47 | 1.60 | 1.60 | 1.02 | 1.38 | 1.58 | 0.92 | 0.77 | 1.50 | 1.16 |
| 39.192 | 3-Cyclopentylpropionic acid, 2-dimethylaminoethyl ester | 1.57 | 1.93 | 2.35 | 1.59 | 2.85 | 1.85 | 1.62 | 1.67 | 1.77 | 1.79 | 2.47 | 2.65 | 2.24 |
| 39.211 | Fumaric acid, 2-dimethylaminoethyl nonyl ester | nd | nd | nd | nd | nd | nd | nd | nd | nd | nd | nd | nd | nd |
| 40.759 | 2-Phenyl-1,3-dioxan-5-yl 9,12,15-octadecatrienoate | 0.58 | 0.59 | 0.74 | 0.71 | 0.96 | 0.60 | 0.50 | 0.71 | 0.81 | 0.67 | 0.75 | 0.93 | 0.98 |
| 41.611 | 2-Monopalmitin | 1.21 | 2.42 | 1.30 | 2.44 | 0.71 | 1.45 | 1.46 | 1.20 | 0.50 | 0.85 | 1.19 | 1.31 | 0.23 |
| 44.068 | Squalene | 0.22 | 0.15 | nd | nd | nd | 0.09 | 0.17 | nd | 0.15 | nd | nd | nd | nd |
| 44.402 | Monoolein | nd | nd | nd | nd | nd | nd | nd | 0.14 | nd | nd | nd | 0.31 | nd |
| 44.745 | β-Monolinolein | 1.94 | 2.42 | 2.52 | 2.79 | 3.08 | 2.56 | 2.27 | 2.16 | 1.49 | 0.60 | 2.26 | 1.45 | 1.96 |
| 46.754 | Δ-Tocopherol | 0.89 | 0.47 | 1.20 | 1.46 | 0.71 | 1.03 | 0.81 | 1.01 | 1.01 | 0.62 | 0.99 | 0.61 | 1.44 |
| 47.811 | γ-Tocopherol | 2.80 | 2.62 | 1.96 | 2.81 | 2.31 | 2.71 | 2.46 | 2.87 | 4.11 | 2.62 | 1.78 | 1.59 | 2.23 |
| 47.911 | Stigmastan-3,5-diene | nd | 0.26 | 0.19 | 0.28 | 0.24 | 0.30 | 0.19 | 0.07 | 0.20 | 0.22 | 1.11 | 0.22 | 0.29 |
| 48.597 | α-Tocopherol | 0.45 | 0.36 | nd | nd | 0.20 | nd | nd | nd | nd | nd | 0.22 | 0.58 | nd |
| 50.430 | Campesterol | 2.18 | 1.40 | 2.37 | 1.97 | 1.77 | 2.51 | 5.17 | 2.60 | 2.04 | 1.19 | 0.70 | 1.20 | 1.68 |
| 50.773 | Stigmasterol | 2.68 | 2.91 | 4.65 | 2.03 | 2.45 | 3.16 | 4.90 | 2.20 | 4.40 | 2.01 | 2.54 | 1.86 | 1.98 |
| 51.626 | γ-Sitosterol | 5.61 | 6.01 | 4.58 | 7.16 | 4.80 | 7.20 | 5.97 | 6.61 | 10.54 | 5.17 | 6.29 | 5.63 | 4.40 |

**S1 Table.** Continue...

| ***R_t_*** | **Compound Identification** | **SBR41** | **SBR42** | **SBR43** | **SBR44** | **SBR45** | **SBR46** | **SBR47** | **SBR48** | **SBR49** | **SBR50** | **SBR51** | **SBR52** | **SBR53** |
| --- | --- | --- | --- | --- | --- | --- | --- | --- | --- | --- | --- | --- | --- | --- |
| 5.044 | Methyl pyruvate | 0.55 | 0.53 | 0.93 | 0.26 | 0.57 | 0.76 | 0.37 | 0.57 | 0.39 | 0.53 | 0.14 | nd | 0.45 |
| 7.763 | Dihydroxyacetone | nd | nd | nd | nd | nd | nd | nd | nd | nd | nd | nd | nd | nd |
| 8.068 | 2,4-Dihydroxy-2,5-dimethyl-3(2H)-furan-3-one | 1.81 | 3.13 | 2.18 | 2.15 | 2.87 | 3.29 | 0.94 | 2.34 | 0.26 | 1.25 | 1.91 | 2.35 | 1.60 |
| 8.254 | Pyranone | nd | nd | nd | nd | nd | nd | nd | nd | nd | nd | nd | nd | nd |
| 11.530 | 2-Hydroxy-γ-butyrolactone | 0.13 | 0.67 | 0.52 | 0.53 | 0.32 | 0.46 | 0.33 | 0.30 | 0.13 | 0.09 | 0.32 | 0.30 | 0.25 |
| 11.887 | Maltol | 0.06 | 0.95 | 1.21 | 0.97 | 0.36 | 0.65 | 0.59 | 0.90 | 0.71 | 0.45 | 0.45 | 0.57 | 0.26 |
| 12.025 | l-Alanine, n-propargyloxycarbonyl-, hexyl ester | nd | nd | nd | nd | nd | nd | nd | nd | 0.12 | nd | nd | nd | nd |
| 12.168 | 4,5-Diamino-2-hydroxypyrimidine | nd | nd | nd | nd | nd | nd | nd | nd | nd | 0.16 | nd | nd | nd |
| 15.101 | Serine, acetate | nd | nd | 0.15 | nd | nd | nd | nd | nd | nd | nd | nd | nd | nd |
| 15.130 | l-Alanine, N-methoxycarbonyl-, heptyl ester | nd | nd | nd | nd | nd | nd | nd | nd | nd | nd | nd | nd | nd |
| 15.144 | α-Methylacetoacetic ester | nd | nd | nd | nd | nd | nd | nd | nd | nd | nd | nd | nd | nd |
| 15.316 | Propanoic acid, 3-(acetylthio)-2-methyl- | nd | nd | nd | nd | nd | nd | nd | nd | nd | nd | nd | nd | nd |
| 17.211 | 5-Hydroxymethylfurfural | 0.35 | 0.44 | 3.36 | 3.36 | 2.14 | 2.49 | 2.28 | 3.52 | 4.04 | 2.59 | 2.83 | 3.53 | 2.33 |
| 17.625 | 4-Vinylguaiacol | 0.04 | 0.02 | nd | nd | nd | nd | nd | nd | nd | nd | nd | nd | nd |
| 19.440 | 2,3-Dimethoxyphenol | 0.46 | 0.14 | 0.59 | 0.49 | 0.44 | 0.26 | 0.44 | 0.43 | 0.72 | 0.37 | 0.73 | 0.57 | 0.74 |
| 22.916 | Sucrose | 15.27 | 19.04 | 16.98 | 16.99 | 13.37 | 16.09 | 13.67 | 18.53 | 13.02 | 13.24 | 14.32 | 15.84 | 12.02 |
| 23.759 | 3,5-Dimethoxyacetophenone | 0.40 | 0.26 | 0.66 | 0.18 | 0.15 | 0.31 | 0.11 | 0.11 | 0.28 | 0.22 | 0.29 | 0.18 | 0.13 |
| 26.406 | Methyl palmitate | 0.67 | 0.60 | 0.75 | 0.88 | 0.58 | 0.54 | 0.36 | 1.02 | 0.82 | 0.70 | 0.51 | 0.90 | 1.01 |
| 26.716 | 3-Deoxy-d-mannoic lactone | nd | nd | nd | nd | nd | nd | nd | nd | nd | nd | nd | nd | nd |
| 26.878 | d-Mannose | nd | nd | nd | nd | nd | nd | nd | nd | nd | nd | nd | nd | nd |
| 27.059 | 3-O-Methylhexose | 8.53 | 1.30 | 6.55 | 9.54 | 18.72 | 14.84 | 24.89 | 7.53 | 12.89 | 10.76 | 19.87 | 9.00 | 6.78 |
| 27.535 | Palmitic acid | 7.56 | 4.73 | 8.69 | 9.54 | 7.06 | 6.85 | 5.82 | 7.64 | 8.37 | 8.69 | 7.74 | 8.49 | 10.76 |
| 30.273 | 13-Octadecenoic acid, methyl ester | 0.48 | 0.46 | 0.40 | 0.42 | 0.51 | 0.82 | 0.71 | 0.79 | 0.46 | 0.72 | 0.46 | 0.66 | 0.90 |
| 30.621 | Methyl linoleate | 2.45 | 1.99 | 1.74 | 1.91 | 2.22 | 2.53 | 1.74 | 2.59 | 2.16 | 1.96 | 1.83 | 2.80 | 3.93 |
| 31.678 | Oleic acid | 3.90 | 3.40 | 4.92 | 5.32 | 3.99 | 6.02 | 5.64 | 5.02 | 3.78 | 5.39 | 3.87 | 4.72 | 5.45 |
| 32.111 | Linoleic acid | 17.58 | 21.47 | 19.09 | 20.21 | 16.85 | 15.70 | 15.27 | 17.12 | 17.90 | 17.24 | 18.21 | 18.81 | 21.15 |
| 32.840 | Linolenic acid | 0.71 | 1.48 | 1.08 | 1.23 | 0.71 | 0.81 | 0.86 | 0.80 | 1.28 | 0.87 | 0.89 | 0.65 | 0.84 |
| 34.197 | Fumaric acid, decyl 2-dimethylaminoethyl ester | 0.61 | 0.70 | 0.78 | 0.53 | 0.57 | 0.43 | 0.48 | 0.55 | 0.63 | 0.65 | 0.58 | 0.57 | 0.76 |
| 34.240 | Octanoic acid, 2-dimethylaminoethyl ester | nd | nd | nd | nd | nd | nd | nd | nd | nd | nd | nd | nd | nd |
| 36.021 | Glycidyl palmitate | 0.37 | 0.29 | 0.37 | 0.28 | 0.31 | 0.25 | 0.26 | 0.38 | 0.37 | 0.38 | 0.26 | 0.37 | 0.37 |
| 36.635 | Palmidrol | 0.22 | 0.48 | 0.33 | 0.09 | 0.19 | 0.18 | 0.27 | 0.35 | 0.27 | 0.31 | 0.25 | 0.26 | 0.32 |
| 37.064 | 4,5-Dihydro-2-[(8Z,11Z)-8,11-heptadecadienyl]oxazole | 1.21 | 1.95 | 1.40 | 0.33 | 0.81 | 0.40 | 0.77 | 1.23 | 1.56 | 1.09 | 1.61 | 1.25 | 1.45 |
| 39.192 | 3-Cyclopentylpropionic acid, 2-dimethylaminoethyl ester | 2.24 | 1.97 | 2.47 | 1.66 | 1.58 | 1.46 | 1.55 | 1.82 | 1.76 | 1.93 | 1.92 | 1.63 | 2.17 |
| 39.211 | Fumaric acid, 2-dimethylaminoethyl nonyl ester | nd | nd | nd | nd | nd | nd | nd | nd | nd | nd | nd | nd | nd |
| 40.759 | 2-Phenyl-1,3-dioxan-5-yl 9,12,15-octadecatrienoate | 0.80 | 0.79 | 0.75 | 0.62 | 0.62 | 0.53 | 0.56 | 0.90 | 0.72 | 0.83 | 0.71 | 0.76 | 0.94 |
| 41.611 | 2-Monopalmitin | 3.12 | 3.15 | 2.36 | 1.68 | 2.91 | 1.84 | 1.92 | 2.35 | 3.28 | 3.21 | 1.70 | 3.21 | 3.40 |
| 44.068 | Squalene | 0.11 | 0.13 | 0.07 | 0.12 | 0.12 | nd | 0.13 | 0.20 | 0.15 | 0.74 | 0.12 | 0.15 | 0.11 |
| 44.402 | Monoolein | nd | nd | nd | nd | nd | nd | nd | nd | nd | nd | nd | nd | nd |
| 44.745 | β-Monolinolein | 2.23 | 4.03 | 2.28 | 2.76 | 3.25 | 2.93 | 2.41 | 2.87 | 3.00 | 2.01 | 1.85 | 2.36 | 1.89 |
| 46.754 | Δ-Tocopherol | 0.60 | 1.17 | 0.80 | 1.13 | 0.54 | 0.92 | 0.34 | 0.78 | 0.60 | 0.51 | 0.33 | 0.71 | 0.35 |
| 47.811 | γ-Tocopherol | 2.56 | 3.05 | 2.15 | 2.76 | 2.57 | 2.74 | 2.09 | 2.55 | 3.80 | 2.01 | 2.32 | 3.32 | 2.43 |
| 47.911 | Stigmastan-3,5-diene | 0.29 | 0.41 | 0.24 | 0.40 | 0.36 | 0.44 | 0.38 | 0.36 | 0.45 | 0.36 | 0.28 | 0.45 | 0.43 |
| 48.597 | α-Tocopherol | 0.47 | 0.17 | nd | 0.32 | 0.37 | 0.45 | 0.25 | 0.56 | 0.85 | 0.07 | 0.27 | 0.71 | 0.60 |
| 50.430 | Campesterol | 3.57 | 2.49 | 1.72 | 0.96 | 1.26 | 0.86 | 1.90 | 1.86 | 1.11 | 2.50 | 1.21 | 1.63 | 1.96 |
| 50.773 | Stigmasterol | 8.08 | 2.95 | 2.11 | 1.94 | 3.10 | 3.06 | 1.77 | 2.41 | 1.94 | 6.71 | 2.62 | 1.64 | 3.86 |
| 51.626 | γ-Sitosterol | 5.09 | 5.82 | 6.38 | 4.38 | 5.22 | 4.41 | 5.43 | 5.35 | 6.71 | 5.47 | 4.77 | 6.39 | 5.81 |

^a^ Reported resistant soybean accessions were highlighted in green, susceptible accessions were highlighted in yellow; unhighlighted accessions were unknown.
