# Supplementary material for: Phytochemical profiling of soybean genotypes using GC-MS and UHPLC-DAD/MS
Source: PLoS One. 2024 Aug 15;19(8):e0308489. doi: 10.1371/journal.pone.0308489 (PMC11326653; doi:10.1371/journal.pone.0308489)
Supplement: S2 Table — (DOCX) [file pone.0308489.s002.docx]

**Supporting Information (S2 Table)**

**Phytochemical profiling of soybean genotypes with different reactions to soybean diseases using GC-MS and UHPLC/DAD-MS**

Shuxian Li^1*^, Mei Wang^2^, Joseph Lee^3^

**^1^** United States Department of Agriculture, Agricultural Research Service (USDA, ARS), Crop Genetics Research Unit, Stoneville, MS 38776, USA.

**^2^** USDA, ARS, Natural Products Utilization Research Unit, University, MS 38677, USA.

**^3^** National Center for Natural Products Research, School of Pharmacy, University of Mississippi, University, MS 38677, USA.

**S2 Table.** Compositional data expressed as different groups of compounds used for PCA. ^a^

| **Sample** | **Ester** | **Ketone** | **Heterocyclic Compound** | **Amine** | **Carboxylic**  **Acid** | **Aldehyde** | **Phenolic**  **Compound** | **Sugar**  **Moiety** | **Lactone** | **Amide** | **Triterpene** | **Tocopherol** | **Phytosterol** |
| --- | --- | --- | --- | --- | --- | --- | --- | --- | --- | --- | --- | --- | --- |
| SBR1 | 5.56 | 2.21 | 0.95 | 0.00 | 24.45 | 3.95 | 1.46 | 45.52 | 0.00 | 0.00 | 0.00 | 5.40 | 10.51 |
| SBR2 | 4.84 | 1.72 | 0.00 | 0.00 | 32.76 | 3.45 | 1.68 | 32.28 | 0.00 | 0.00 | 0.00 | 4.95 | 18.32 |
| SBR3 | 2.97 | 1.37 | 1.95 | 0.00 | 28.35 | 4.23 | 0.00 | 33.28 | 0.00 | 0.00 | 0.00 | 8.38 | 19.49 |
| SBR4 | 2.65 | 2.91 | 1.86 | 0.00 | 22.86 | 7.19 | 0.00 | 31.48 | 1.87 | 0.00 | 0.00 | 5.44 | 23.75 |
| SBR5 | 2.47 | 3.35 | 2.36 | 0.00 | 27.55 | 6.04 | 0.00 | 37.78 | 0.00 | 0.00 | 0.00 | 5.23 | 15.22 |
| SBR6 | 6.17 | 2.67 | 2.32 | 0.00 | 25.09 | 5.68 | 0.00 | 32.85 | 0.00 | 0.00 | 0.00 | 6.39 | 18.82 |
| SBR7 | 4.62 | 3.27 | 2.66 | 0.00 | 25.85 | 7.51 | 0.00 | 34.67 | 1.01 | 0.00 | 0.00 | 3.05 | 17.37 |
| SBR8 | 5.56 | 4.38 | 2.97 | 0.71 | 22.02 | 8.29 | 0.00 | 37.22 | 0.00 | 0.00 | 0.00 | 4.28 | 14.57 |
| SBR9 | 5.73 | 3.02 | 2.03 | 1.03 | 27.75 | 4.84 | 0.00 | 34.22 | 0.00 | 0.00 | 0.00 | 4.92 | 16.47 |
| SBR10 | 4.10 | 2.39 | 1.77 | 0.00 | 27.75 | 3.39 | 0.00 | 40.19 | 0.00 | 0.00 | 0.00 | 4.79 | 15.61 |
| SBR11 | 6.07 | 2.67 | 1.91 | 0.00 | 29.04 | 4.89 | 0.91 | 32.22 | 0.00 | 0.00 | 0.00 | 4.96 | 17.34 |
| SBR12 | 9.96 | 1.77 | 0.00 | 0.00 | 40.80 | 3.02 | 0.97 | 26.03 | 0.00 | 0.00 | 0.00 | 4.25 | 12.85 |
| SBR13 | 8.39 | 2.08 | 2.08 | 0.00 | 35.38 | 4.04 | 0.00 | 26.59 | 0.00 | 0.00 | 0.00 | 6.58 | 13.79 |
| SBR14 | 11.98 | 0.80 | 1.32 | 0.00 | 33.43 | 2.85 | 0.77 | 34.91 | 0.00 | 0.00 | 0.00 | 4.14 | 9.23 |
| SBR15 | 7.51 | 1.02 | 1.38 | 0.00 | 30.54 | 3.13 | 0.00 | 32.38 | 0.00 | 0.00 | 0.00 | 5.86 | 18.17 |
| SBR16 | 7.61 | 2.07 | 1.45 | 0.00 | 30.89 | 4.69 | 0.00 | 31.40 | 0.00 | 0.00 | 0.00 | 4.08 | 17.00 |
| SBR17 | 7.13 | 1.81 | 1.37 | 0.00 | 29.70 | 4.95 | 0.00 | 34.37 | 0.00 | 1.03 | 0.00 | 5.20 | 14.45 |
| SBR18 | 7.21 | 1.60 | 1.04 | 0.00 | 34.19 | 2.40 | 0.65 | 33.03 | 0.00 | 0.00 | 0.00 | 4.78 | 15.11 |
| SBR19 | 9.05 | 0.58 | 1.98 | 0.00 | 37.01 | 2.15 | 0.00 | 35.08 | 0.00 | 1.08 | 0.00 | 4.74 | 7.54 |
| SBR20 | 9.95 | 2.42 | 0.00 | 0.00 | 32.22 | 1.54 | 0.00 | 32.70 | 0.00 | 0.00 | 0.00 | 3.86 | 16.65 |
| SBR21 | 15.58 | 2.22 | 1.32 | 1.21 | 27.89 | 4.06 | 0.53 | 29.75 | 0.00 | 0.00 | 0.00 | 3.84 | 13.61 |
| SBR22 | 10.59 | 1.91 | 0.00 | 0.00 | 28.93 | 3.95 | 0.68 | 37.87 | 0.00 | 0.00 | 0.87 | 2.14 | 12.48 |
| SBR23 | 13.87 | 0.91 | 1.63 | 0.00 | 36.64 | 1.40 | 0.00 | 30.36 | 0.00 | 0.00 | 0.00 | 4.13 | 11.07 |
| SBR24 | 12.87 | 0.72 | 2.81 | 0.00 | 30.02 | 2.46 | 0.00 | 39.96 | 0.00 | 0.00 | 0.00 | 3.71 | 7.46 |
| SBR26 | 9.91 | 0.69 | 2.43 | 0.00 | 35.44 | 6.42 | 0.00 | 30.63 | 0.00 | 0.53 | 0.00 | 5.07 | 8.13 |
| SBR27 | 13.71 | 2.47 | 0.91 | 0.00 | 35.49 | 1.74 | 0.42 | 33.54 | 0.00 | 0.00 | 0.00 | 4.42 | 8.87 |
| SBR28 | 12.77 | 0.69 | 0.89 | 0.00 | 34.16 | 1.35 | 0.89 | 32.65 | 0.00 | 0.00 | 0.00 | 4.42 | 12.56 |
| SBR29 | 12.52 | 0.40 | 1.38 | 0.00 | 30.36 | 3.57 | 0.00 | 35.93 | 0.00 | 0.00 | 0.00 | 3.88 | 11.59 |
| SBR30 | 11.47 | 11.47 | 3.53 | 0.00 | 33.65 | 2.62 | 1.56 | 28.91 | 0.00 | 0.00 | 0.00 | 3.66 | 13.43 |
| SBR31 | 12.52 | 2.35 | 1.36 | 0.00 | 29.78 | 2.71 | 0.00 | 32.15 | 0.00 | 0.00 | 0.00 | 4.80 | 12.53 |
| SBR32 | 9.59 | 0.75 | 0.00 | 0.00 | 33.20 | 3.15 | 0.89 | 33.16 | 0.00 | 0.00 | 0.00 | 3.84 | 11.50 |
| SBR33 | 10.94 | 1.90 | 2.61 | 0.00 | 34.49 | 1.98 | 0.46 | 28.70 | 0.00 | 0.55 | 0.00 | 4.14 | 14.24 |
| SBR34 | 10.76 | 0.79 | 1.37 | 0.00 | 32.71 | 3.32 | 0.00 | 24.69 | 0.00 | 0.00 | 0.00 | 4.34 | 21.30 |
| SBR35 | 11.07 | 1.66 | 1.30 | 0.00 | 40.04 | 2.10 | 0.00 | 22.73 | 0.00 | 0.00 | 0.00 | 4.56 | 13.41 |
| SBR36 | 12.14 | 0.00 | 2.79 | 0.00 | 38.24 | 0.65 | 0.00 | 17.86 | 0.00 | 0.00 | 0.00 | 6.56 | 21.76 |
| SBR37 | 9.02 | 0.70 | 5.34 | 0.00 | 33.48 | 5.38 | 0.75 | 30.23 | 0.00 | 0.00 | 0.00 | 3.98 | 10.29 |
| SBR38 | 14.21 | 1.11 | 4.74 | 0.00 | 35.41 | 1.67 | 0.00 | 26.20 | 0.00 | 0.00 | 0.00 | 3.44 | 13.21 |
| SBR39 | 12.78 | 0.00 | 2.29 | 0.00 | 36.49 | 1.32 | 0.00 | 32.15 | 0.00 | 0.00 | 0.00 | 3.63 | 10.72 |
| SBR40 | 11.41 | 0.00 | 5.67 | 0.00 | 34.76 | 2.55 | 0.00 | 28.51 | 0.00 | 0.00 | 0.00 | 4.99 | 12.10 |
| SBR41 | 11.65 | 0.45 | 2.03 | 0.00 | 33.38 | 0.00 | 0.51 | 26.71 | 0.00 | 0.00 | 0.00 | 3.55 | 18.79 |
| SBR42 | 15.61 | 0.00 | 4.75 | 0.00 | 36.13 | 0.51 | 0.00 | 23.66 | 0.00 | 0.56 | 0.00 | 4.90 | 13.11 |
| SBR43 | 12.30 | 1.30 | 3.74 | 0.00 | 37.28 | 3.71 | 0.65 | 25.97 | 0.00 | 0.00 | 0.00 | 3.26 | 11.27 |
| SBR44 | 12.23 | 0.57 | 2.31 | 0.00 | 39.15 | 3.60 | 0.52 | 28.38 | 0.00 | 0.00 | 0.00 | 4.16 | 8.21 |
| SBR45 | 13.29 | 0.00 | 3.51 | 0.00 | 31.04 | 2.32 | 0.48 | 34.80 | 0.00 | 0.00 | 0.00 | 3.78 | 10.79 |
| SBR46 | 12.87 | 0.00 | 3.61 | 0.00 | 32.23 | 2.73 | 0.00 | 33.93 | 0.00 | 0.00 | 0.00 | 4.50 | 9.61 |
| SBR47 | 11.19 | 0.35 | 0.78 | 0.00 | 29.94 | 2.48 | 0.48 | 41.85 | 0.00 | 0.00 | 0.00 | 2.64 | 10.29 |
| SBR48 | 15.28 | 0.00 | 2.59 | 0.00 | 33.89 | 3.90 | 0.48 | 28.89 | 0.00 | 0.00 | 0.00 | 4.31 | 10.66 |
| SBR49 | 14.45 | 0.00 | 0.77 | 0.00 | 34.29 | 4.42 | 0.79 | 28.36 | 0.00 | 0.00 | 0.00 | 5.74 | 9.96 |
| SBR50 | 13.54 | 0.00 | 1.86 | 0.00 | 35.24 | 2.84 | 0.00 | 26.25 | 0.00 | 0.00 | 0.81 | 2.76 | 16.07 |
| SBR51 | 10.82 | 0.00 | 2.42 | 0.00 | 33.18 | 3.05 | 0.79 | 36.85 | 0.00 | 0.00 | 0.00 | 2.86 | 9.30 |
| SBR52 | 13.98 | 0.00 | 3.16 | 0.00 | 35.40 | 3.82 | 0.62 | 26.92 | 0.00 | 0.00 | 0.00 | 5.14 | 10.96 |
| SBR53 | 16.62 | 0.00 | 1.73 | 0.00 | 40.86 | 2.52 | 0.80 | 20.32 | 0.00 | 0.00 | 0.00 | 3.66 | 13.04 |

^a^ Reported resistant soybean accessions were highlighted in green, susceptible accessions were highlighted in yellow; unhighlighted accessions were unknown.
